# Chromatin Dynamics and the RNA Exosome Function in Concert to Regulate Transcriptional Homeostasis

**DOI:** 10.1016/j.celrep.2015.10.030

**Published:** 2015-11-12

**Authors:** Mayuri Rege, Vidya Subramanian, Chenchen Zhu, Tsung-Han S. Hsieh, Assaf Weiner, Nir Friedman, Sandra Clauder-Münster, Lars M. Steinmetz, Oliver J. Rando, Laurie A. Boyer, Craig L. Peterson

**Affiliations:** 1 Program in Molecular Medicine, University of Massachusetts Medical School, Worcester, MA 01605, USA; 2 Department of Biology, Massachusetts Institute of Technology, Cambridge, MA 02139, USA; 3 Genome Biology Unit, European Molecular Biology Laboratory, Heidelberg 69117, Germany; 4 Department of Biochemistry and Molecular Pharmacology, University of Massachusetts Medical School, Worcester, MA 01605, USA; 5 School of Computer Science and Engineering, The Hebrew University, Jerusalem 91904, Israel; 6 Alexander Silberman Institute of Life Sciences, The Hebrew University, Jerusalem 91904, Israel; 7 Department of Genetics, Stanford University School of Medicine, Stanford, CA 94305, USA

## Abstract

The histone variant H2A.Z is a hallmark of nucleosomes flanking promoters of protein-coding genes and is often found in nucleosomes that carry lysine 56-acetylated histone H3 (H3-K56Ac), a mark that promotes replication-independent nucleosome turnover. Here, we find that H3-K56Ac promotes RNA polymerase II occupancy at many protein-coding and noncoding loci, yet neither H3-K56Ac nor H2A.Z has a significant impact on steady-state mRNA levels in yeast. Instead, broad effects of H3-K56Ac or H2A.Z on RNA levels are revealed only in the absence of the nuclear RNA exosome. H2A.Z is also necessary for the expression of divergent, promoter-proximal non-coding RNAs (ncRNAs) in mouse embryonic stem cells. Finally, we show that H2A.Z functions with H3-K56Ac to facilitate formation of chromosome interaction domains (CIDs). Our study suggests that H2A.Z and H3-K56Ac work in concert with the RNA exosome to control mRNA and ncRNA expression, perhaps in part by regulating higher-order chromatin structures.

## Graphical Abstract

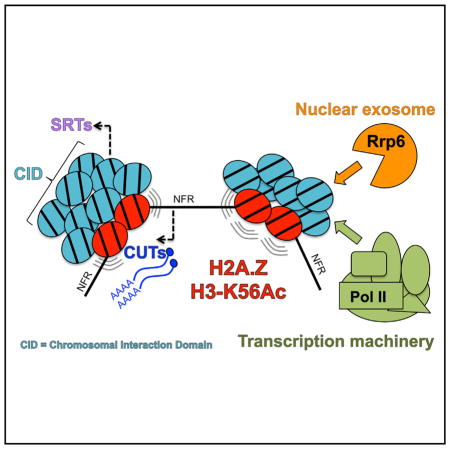

## INTRODUCTION

Nucleosomes that flank gene regulatory elements in eukaryotes exhibit rapid, replication-independent nucleosome replacement (Dion et al., 2007; Rufiange et al., 2007). This enhanced nucleosome turnover occurs at nucleosomes carrying the histone variant H2A.Z and is slowed in the absence of histone H3 lysine 56 acetylation (H3-K56Ac) (Albert et al., 2007; Kaplan et al., 2008; Raisner et al., 2005; Rufiange et al., 2007). The dynamic nature of these nucleosomes has contributed to the prevailing view that these chromatin features may generally promote transcription. However, previous studies have failed to reveal extensive transcription roles for either H3-K56Ac orH2A.Z (Lenstra etal., 2011;Mizuguchi et al., 2004), and thus their contribution to transcription remains unclear.

In addition to harboring dynamic nucleosomes, eukaryotic promoter regions are commonly bi-directional in nature, with divergent noncoding RNAs (ncRNAs) and mRNAs expressed from different promoters that share a common nucleosome free region (NFR) (Neil et al., 2009; Xu et al., 2009). In yeast, many divergently transcribed ncRNAs are cryptic unstable transcripts (CUTs) that are 5′ capped and polyadenylated, with a median length of 400 bp. Normally, CUTs are rapidly degraded because they contain binding motifs for the Nrd1/Nab3/Sen1 (NNS) termination machinery which, in turn, promotes recruitment of the RNA exosome (Arigo et al., 2006; Schulz et al., 2013; Thiebaut et al., 2006). Consequently, inactivation of the nuclear exosome subunit, Rrp6, is necessary to monitor changes in CUT transcription. Rrp6 is a 3′-5′ exonuclease that also targets ncRNAs and unspliced pre-mRNAs for degradation (Schneider et al., 2012), facilitates processing of small nuclear/small nucleolar RNAs (Gudipati et al., 2012), promotes fidelity of mRNA termination (Schaeffer and van Hoof, 2011), and may play a more general surveillance role that governs nuclear mRNA levels (Schmid et al., 2012). Whether H2A.Z or H3-K56Ac regulates expression of ncRNAs has not been thoroughly addressed.

CUTs represent but one of several classes of ncRNAs found in yeast. Another class of ncRNAs of particular interest comprises Ssu72 restricted transcripts (SRTs), which accumulate in the absence of the transcription termination factor Ssu72 and also seem to be targeted by the exosome (Tan-Wong et al., 2012). Of the 605 SRTs, 135 are promoter associated, while many are found at 3′ ends of convergent gene pairs and may reflect aberrant termination events (Tan-Wong et al., 2012). Ssu72 is a subunit of the RNA 3′ end-processing machinery that is associated with the RNAPII C-terminal domain (CTD) (Dichtl et al., 2002), and it functions as a CTD Ser5 phosphatase during termination (Krishnamurthy et al., 2004). Ssu72 also functionally interacts with other components of the transcription pre-initiation machinery (e.g., TFIIB) (Pappas and Hampsey, 2000) and may facilitate interactions between the 5′ and 3′ ends of genes, promoting gene “loops” (Tan-Wong et al., 2012). Intriguingly, the strongest genetic interactions of Ssu72 are with multiple subunits of SWR-C, an ATP-dependent chromatin remodeling complex that deposits H2A.Z at 5′ and 3′ ends of genes, implying that they may function together to regulate SRT expression and/or 3D genome interactions (Collins et al., 2007; Fiedler et al., 2009).

Here, we present evidence that H2A.Z and H3-K56Ac are both global, positive regulators of ncRNA expression in yeast and that H2A.Z also enhances the expression of a subset of divergent ncRNAs in mouse embryonic stem cells (mESCs), indicating a conserved role for H2A.Z in regulating divergent transcription. We also show that H3-K56Ac has a dramatic effect on RNAPII occupancy at many protein-coding genes, but corresponding changes in mRNA levels are masked by a functional nuclear exosome. Surprisingly, our study also uncovers a repressive role for H2A.Z where it functions together with the nuclear exosome to repress expression of a subset of ncRNAs. Finally, we find that H2A.Z, like H3-K56Ac, contributes to the formation of higher-order chromosome interaction domains (CIDs) that we propose may play a role in the regulation of ncRNA expression.

## RESULTS

### H2A.Z and H3-K56Ac Have Little Apparent Impact on Steady-State RNA Abundance

**Figure 1:**
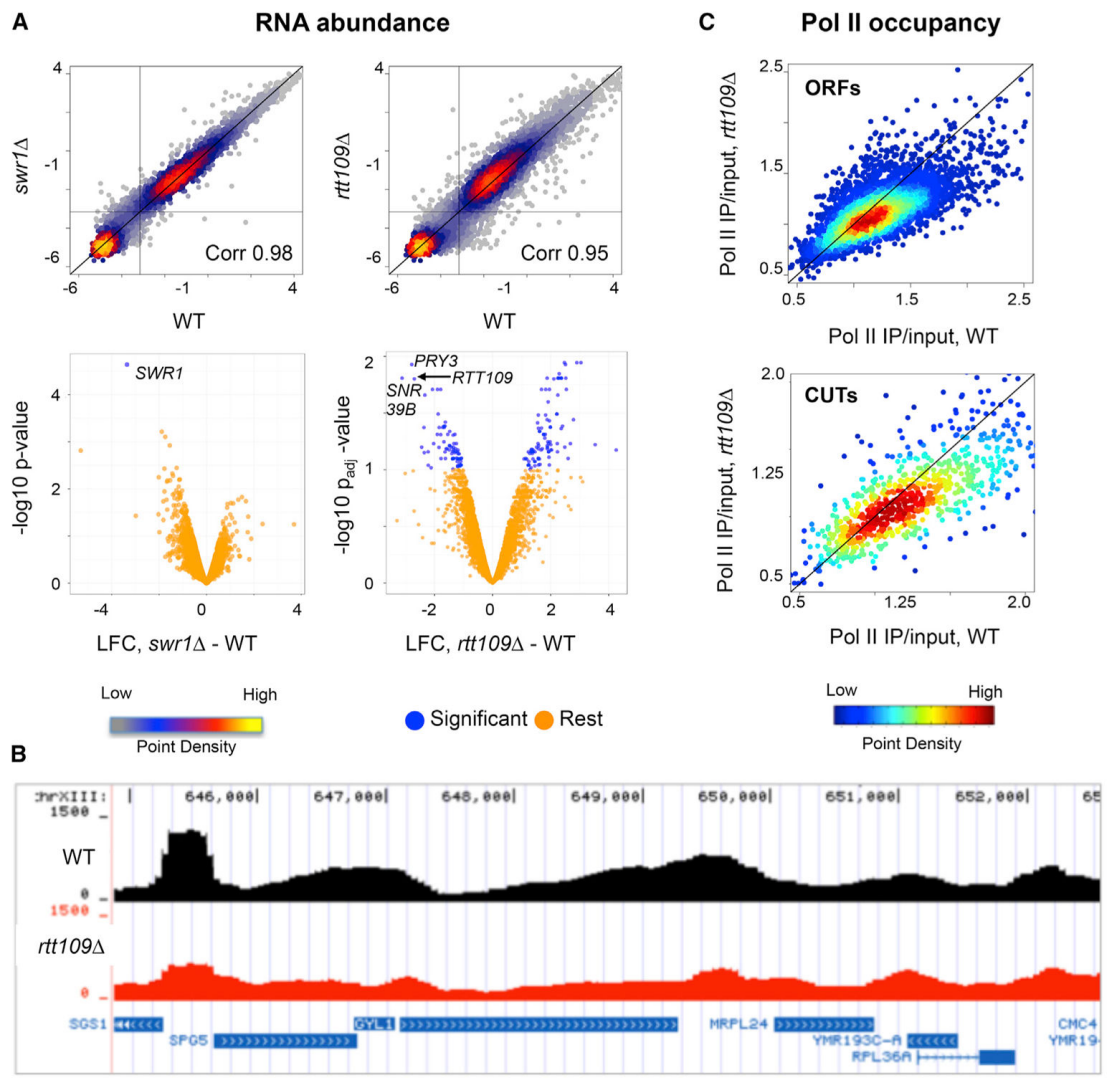
H3-K56Ac Regulates Pol II Recruitment, although RNA Levels Are Less Affected (A) RNA abundance measured by strand-specific tiling microarrays in *swr1Δ* and *rtt109Δ* strains. Density scatterplots (top panels) show median signal intensity values in comparison to wild-type (WT) arrays. The black diagonal line indicates x = y (no change) and the horizontal and vertical lines indicate the noise threshold cut-off. Volcano plots (bottom panels) show the transcripts that change significantly in the mutant compared to WT highlighted in blue (p_adj_ = FDR < 0.1 and log_2_ fold change > 0.59). The y axis shows the p value (without FDR correction) for *swr1Δ* and p_adj_ value (after FDR correction) for *rtt109Δ*. See also Table S1. (B) Representative genome browser view of Pol II ChIP-seq data for the wild-type (black) and *rtt109Δ* (red), normalized to the respective total library read count. (C) Density scatterplots of Pol II IP/input values in the *rtt109Δ* compared to WT at 5171 ORFs (top) and 925 CUTs (bottom). The black line indicates x = y (no change).

In order to monitor the effect of H2A.Z and H3-K56Ac on both coding and noncoding RNA expression, total RNA was isolated from isogenic wild-type and mutant budding yeast strains, and samples were prepared for hybridization to strand-specific DNA tiling arrays that provide high-density coverage of the yeast transcriptome (Castelnuovo et al., 2014; David et al., 2006; Huber et al., 2006). Initial analyses included strains that harbor gene deletions inactivating the SWR-C chromatin remodeling enzyme that deposits H2A.Z (*swr1Δ*), or the Rtt109 histone acetyltransferase that catalyzes H3-K56 acetylation (*rtt109Δ*). Consistent with previous studies, loss of H2A.Z deposition (*swr1Δ*) had little effect on steady-state transcript abundance compared to wild-type (WT) (Mizuguchi et al., 2004), as no transcripts were reduced 1.5-fold or more from the 7,987 total transcripts monitored at a stringent criterion of false discovery rate (FDR) < 0.1. Indeed, even at a reduced stringency (FDR < 0.8), only a few transcripts were reduced 2-fold or more (Figure 1A; Table S1A). Likewise, inactivation of the Rtt109 acetyltransferase had a minor overall effect on the transcriptome, as only 72 transcripts were decreased 1.5-fold or more compared to WT at an FDR < 0.1 (Figure 1A; Table S1B) (Lenstra et al., 2011). The minor effect of H3-K56Ac on RNA levels was surprising given that the enhanced nucleosome dynamics promoted by this histone mark are expected to generally promote transcription. One possibility is that H2A.Z and H3-K56Ac function redundantly to promote transcription. To test this idea, RNA levels were analyzed from the *swr1Δ rtt109Δ* double mutant. Interestingly, 214 transcripts from the 7,987 total were decreased in the double mutant (1.5-fold at FDR < 0.1), consistent with H2A.Z and H3-K56Ac functioning in parallel pathways to promote expression of a small subset of transcripts (Table S1C).

### Functional Interactions between Chromatin Dynamics and the RNA Exosome

As RNA abundance reflects both synthesis and decay of RNA molecules, we sought to probe the transcription process more directly by monitoring genome-wide RNAPII occupancy by chromatin immunoprecipitation sequencing (ChIP-seq) in isogenic WT and *rtt109Δ* strains (Figures 1B and 1C). In contrast to the minor defects observed for mRNA abundance (Figure 1A), the absence of H3-K56Ac led to widespread decreases in RNAPII levels at 567 open reading frames (ORFs) and 184 CUTs (>1.3-fold) (Figure 1C). The discordance between changes in RNAPII and steady-state RNA levels suggests that changes in gene expression may be obscured by compensatory effects on transcript stability/degradation (Haimovich et al., 2013; Sun et al., 2013).

The nuclear exosome is known to regulate the stability of RNAPII transcripts, which include protein-coding transcripts and ncRNAs (Schaeffer and van Hoof, 2011; Schmid et al., 2012). For example, CUTs are typically not detected in RNA samples isolated from strains that contain a functional exosome (Wyers et al., 2005). To test whether the activity of the RNA exosome might be masking the transcriptional effects resulting from loss of H2A.Z or H3-K56Ac, a gene deletion inactivating the nuclear exosome, *rrp6Δ*, was introduced into the *swr1Δ* and *rtt109Δ* strains. Interestingly, inactivation of the nuclear exosome led to a synthetic slow-growth phenotype in combination with either *swr1Δ* or *rtt109Δ* (Figure S1A). We also found consistent slow-growth phenotypes in related *htz1Δ rrp6Δ* and *swr1Δ swc2Δ rrp6Δ* mutants (data not shown) (Halley et al., 2010; Morillo-Huesca et al., 2010).

To assay the effects of H2A.Z and H3-K56Ac on transcription in the absence of confounding effects of exosome-mediated RNA degradation, total RNA was isolated from isogenic WT, *rrp6Δ*, *swr1Δ rrp6Δ*, and *rtt109Δ rrp6Δ* strains, and samples were hybridized to strand-specific DNA tiling arrays. As expected, inactivation of the nuclear exosome caused a dramatic accumulation of CUTs, as well as increased expression of other ncRNAs such as stable unannotated transcripts (SUTs) (Figure S2A) (Neil et al., 2009; Xu et al., 2009). In addition, 985 ORFs were consistently increased in the *rrp6Δ* mutant 1.5-fold or more compared to the wild-type (WT) strain (FDR < 0.1) (Figure S2A; Table S1D). Notably, the increased expression of ORF transcripts in the *rrp6Δ* mutant is not due to defects in transcription termination from upstream loci (Figures S3A and S3B), as the upstream expression level (defined as −100 to TSS) from these ORFs correlates poorly with the downstream expression levels (defined as TSS to +100). Although Rrp6 was shown to promote proper termination at a handful of ORFs and CUTs (n = 7) (Fox et al., 2015), our analyses suggest that this may not be a widespread phenomenon, at least when the Nrd1 termination factor is functional (Schulz et al., 2013). Furthermore, these ORFs are not enriched for spliced transcripts (90 out of 985 have introns), indicating that the increases are not generally due to splicing defects. Loss of Rrp6 also led to a decrease in expression of a similar number of ORFs (n = 851), and these ORFs include the set of ~100 transcripts that were previously shown to be repressed by transcriptional interference from adjacent ncRNAs (Camblong et al., 2007; Castelnuovo et al., 2014). Notably, RNAPII ChIP-seq analysis in the *rrp6Δ* strain did not reveal significant effects of exosome loss on genome-wide RNAPII occupancy, indicating that the observed changes in RNA abundance in the *rrp6Δ* are due to defects in RNA turnover (Figures S2B and S2C) (Fox et al., 2015).

**Figure 2:**
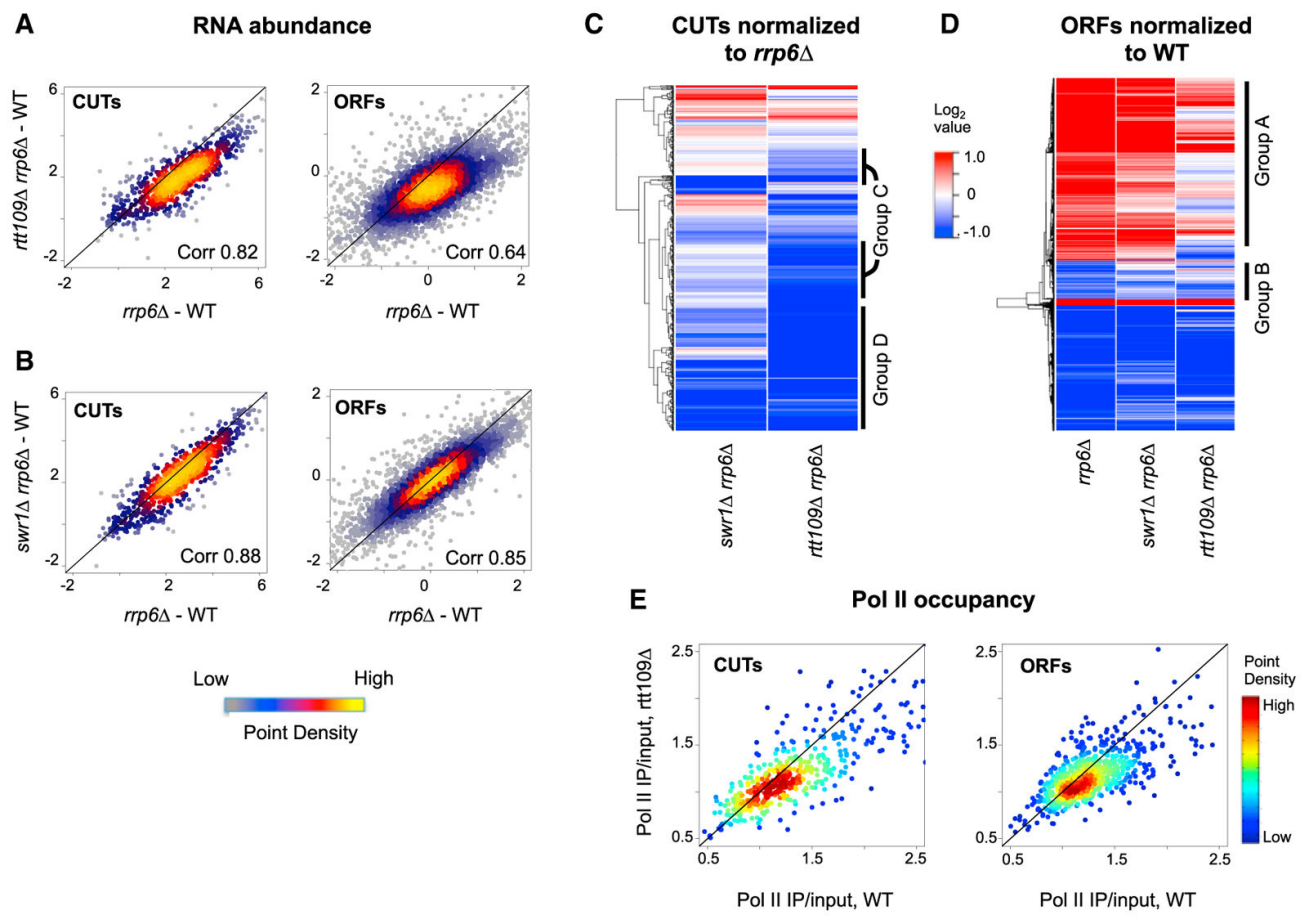
H3-K56Ac and H2A.Z Positively Regulate Transcription in the Absence of the Nuclear Exosome (A and B) RNA abundance measured by strand-specific tiling microarrays in the *rtt109Δ rrp6Δ*, *swr1Δ rrp6Δ*, and *rrp6Δ* mutants normalized to WT. Density scatterplots show log_2_ median intensity values for *rtt109Δ rrp6Δ* (top) and *swr1Δ rrp6Δ* (bottom) plotted against the corresponding value for CUT (left) or ORF (right) transcripts from the *rrp6Δ* strain. The black line indicates x = y (no change). See also Table S1. (C) Heatmap of normalized RNA abundance for CUTs (n = 728) in *rtt109Δ rrp6Δ* and *swr1Δ rrp6Δ* compared to *rrp6Δ*. H3K56Ac-dependent CUTs (group C) as well as H2A.Z- and H3K56Ac-dependent CUTs (group D) are highlighted after hierarchical clustering (Euclidean distance and the complete linkage agglomeration method). CUTs in group C are defined as (1) significantly upregulated in the *rrp6Δ* compared to WT and (2) reduced by > −0.59 LFC in *rtt109Δ rrp6Δ* compared to the *rrp6Δ*. Group D CUTs are defined as (1) significantly upregulated in the *rrp6Δ* compared to WT and (2) reduced by > −0.59 LFC in *rtt109Δ rrp6Δ* as well as *swr1Δ rrp6Δ* compared to the *rrp6Δ*. See also Table S4. (D) Heatmap of normalized RNA abundance for ORFs (n = 1,836) in *rrp6Δ*, *swr1Δ rrp6Δ*, and *rtt109Δ rrp6Δ* compared to WT. Group A and group B ORFs are highlighted after hierarchical clustering (Euclidean distance and the median linkage agglomeration method). Group A ORFs are defined as (1) significantly up-regulated in the *rrp6Δ* compared to WT and (2) reduced by > 0.59 LFC in *rtt109Δ rrp6Δ* compared to the *rrp6Δ*. Group B ORFs are defined as (1) significantly downregulated in *rrp6Δ* compared to WT and (2) increased by > 0.59 LFC in *rtt109Δ rrp6Δ* compared to *rrp6Δ*. See also Table S4. This group includes ORFs subject to transcriptional interference by adjacent CUTs. (E) Density scatterplots of Pol II IP/input values in the *rtt109Δ* compared to wild-type at group C+D CUTs (left) and group A ORFs (right).

By examining the double mutants, we found to our surprise that loss of H3-K56Ac partially suppressed many of the transcriptional changes observed in the *rrp6Δ* strain. Levels of the majority of CUTs were reduced in the *rtt109Δ rrp6Δ* double mutant compared to the *rrp6Δ* strain (Figure 2A, left and Figure 2C, groups C and D), with 394 CUT transcripts showing a decrease in expression of 1.5-fold or more (FDR < 0.1) (Table S1F). Consistent with the hypothesis that loss of Rtt109 specifically affects transcription of these ncRNAs (as opposed to RNA stability, etc.), ORF transcripts that are subject to transcriptional interference by ncRNAs were de-repressed in the *rtt109Δ rrp6Δ* double mutant (Figure 2D, group B; Data S1). In addition to its effects on ncRNA transcription, loss of Rtt109 also affected exosome-sensitive ORFs; those ORFs (n = 985) that showed significantly increased expression in the *rrp6Δ* strain were reduced to near wild-type levels in the *rtt109Δ rrp6Δ* double mutant (Figure 2A, right and Figure 2D, group A; defined in Experimental Procedures). Only 13 of these 985 ORFs overlap with a group of growth-specific genes, indicating that these transcriptional changes are unlikely to be due to indirect effects of growth rate (Airoldi et al., 2009). Notably, the decreased RNA levels in the *rtt109Δ rrp6Δ* strain correlated well with the changes in RNAPII observed in the *rtt109Δ* single mutant, consistent with a direct role for H3-K56Ac in promoting Pol II occupancy at many CUTs and ORFs (Figure 2E). We do note, however, that the extensive changes in CUT RNA levels observed in the *rtt109Δ rrp6Δ* strain are not fully explained by decreases in RNAPII levels. This may reflect a limitation in the resolution of the ChIP-seq dataset or indicate that Rtt109 contributes to CUT expression through additional mechanisms.

Inactivation of the exosome also revealed previously hidden roles for H2A.Z in gene regulation, as the level of a large number of CUTs was decreased by 1.5-fold or more (FDR < 0.1) in the *swr1Δ rrp6Δ* strain compared to the *rrp6Δ* single mutant (Figure 2B, left and Figure 2C, group D). In support of a common function of H2A.Z and H3-K56Ac, the expression of a majority of these H2A.Z-regulated CUTs (n = 202) was also sensitive to loss of H3-K56Ac (Figure 2C, group D). That said, not all CUTs that require H3-K56Ac for full expression are responsive to loss of H2A.Z (Figure 2C, group C; n = 277). This difference may be explained by the observation that CUTs that require H2A.Z for full expression are characterized by lower levels of H3-K56Ac compared to the group of CUTs that are insensitive to H2A.Z loss (Figure S4A; p < 10^−6^). Not only do H3-K56Ac and H2A.Z have similar effects on CUT abundance, but, like *rtt109*Δ, loss of Swr1 activity also affected the expression of ORF transcripts that were upregulated in *rrp6Δ* strains, although again the effects of *swr1Δ* were less dramatic than those due to H3-K56Ac (Figures 2B and 2D, group A; Figure S1B). Furthermore, inactivation of the RNA exosome in the *swr1Δ rtt109Δ* double mutant appeared to be additive with those of the *swr1Δ rrp6Δ* and *rtt109Δ rrp6Δ* double mutants (Figure S1B; Table S1G). Together, these data indicate that both H3-K56Ac and H2A.Z contribute positively to transcription in yeast, with H3-K56Ac generally having a stronger effect than H2A.Z.

### H2A.Z Regulates Divergent ncRNA Expression in Mouse Embryonic Stem Cells

**Figure 3:**
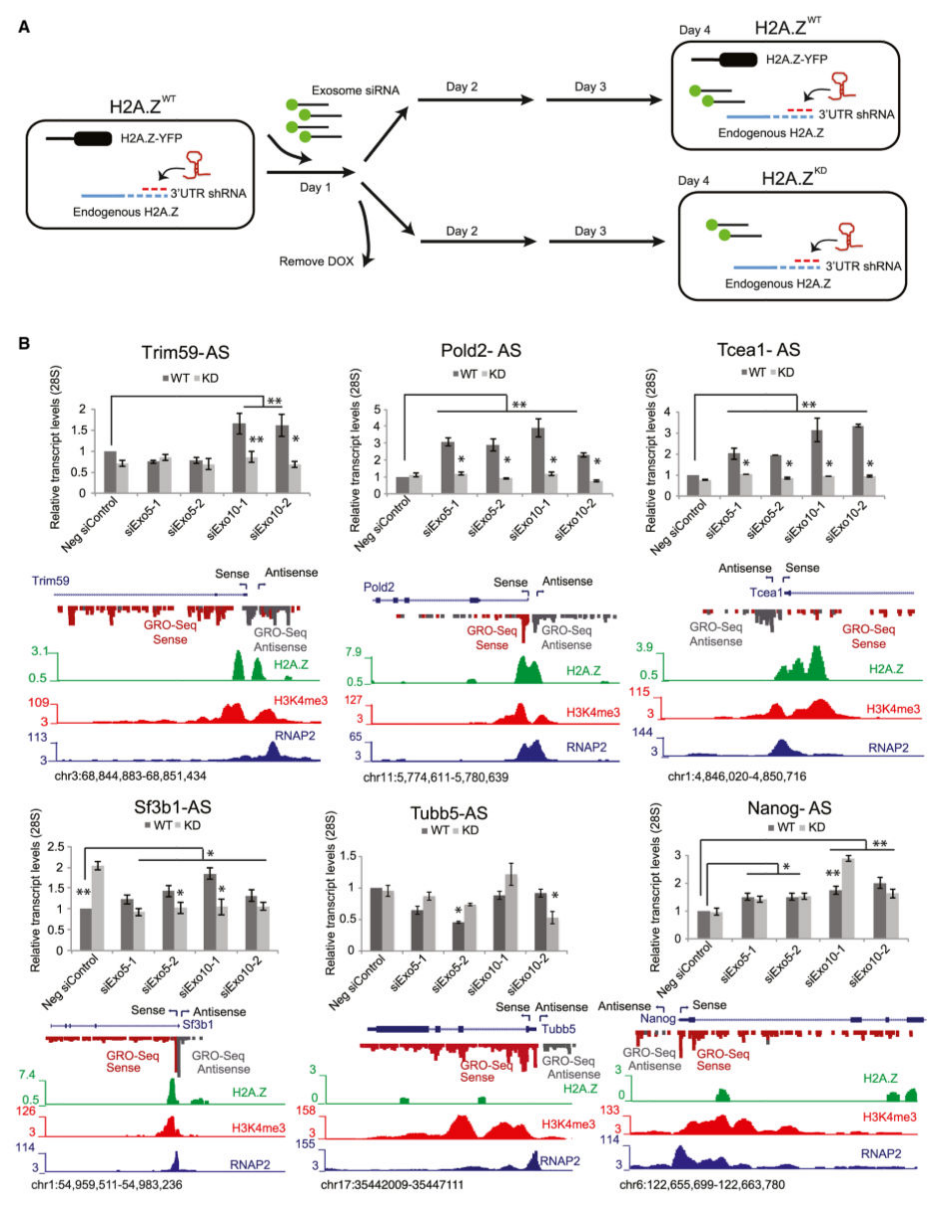
H2A.Z Regulates Divergent ncRNA Expression in Mouse ESCs (A) Schematic representing the transgenic mouse ESC system used to investigate H2A.Z function in regulation of antisense transcription. (B) qRT-PCR representing the relative levels of TSS-associated antisense transcripts (AS) in H2A.Z^WT^ (dark gray) and H2A.Z^KD^ (light gray) mESCs. Transcript levels were normalized to 28S rRNA levels and measured relative to transcript levels in cells treated with non-specific siRNA (Neg siControl). siExo5-1 and 2 and siExo10-1 and 2 refer to two independent siRNAs targeting either exosome component, respectively. Error bars represent standard deviations from a triplicate set of experiments. Trim59S, Pold2, Tcea1, and Sf3b1 are targets of H2A.Z that display bimodal distribution (+1 and −1 nucleosomes) at the TSS. Tubb5 and Nanog are not targets of H2A.Z and serve as controls. Global run-on sequencing (GRO-seq) read density plots (both sense and antisense) from Core et al. (2008), H2A.Z^WT^ (Subramanian et. al., 2013), H3K4me3, and RNAPII (Wamstad et al., 2012) gene tracks of the indicated gene promoter region are depicted below each gene.

Divergently transcribed ncRNAs are also a feature of promoter regions of actively transcribed genes in mouse ESCs, and these transcripts are known substrates for the RNA exosome (Core et al., 2008; Flynn et al., 2011; Seila et al., 2008). Similar to yeast, H2A.Z flanks the nucleosome-free region (NFR) of the majority of actively transcribed genes, while loss of H2A.Z has little effect on the steady-state levels of active genes (Creyghton et al., 2008; Subramanian et al., 2013). Thus, we investigated the role of H2A.Z in regulating the levels of coding region transcripts (sense) or their associated divergent ncRNAs (antisense) at a subset of genes previously shown to produce divergent transcripts (Core et al., 2008; Flynn et al., 2011; Seila et al., 2008) (Figure 3). A transgenic mESC system that harbors a stably integrated Tet-inducible H2A.Z-YFP transgene and short hairpins directed to the endogenous H2A.Z-3′ UTR (Subramanian et al., 2013) was used to measure the effect of H2A.Z on divergent transcription in the presence or absence of the nuclear exosome component Exosc5 (yeast Rrp46) or Exosc10 (yeast Rrp6) (Figure 3A; Figure S5A). Using two independent hairpins, depletion of the exosome components results in a significant increase in anti-sense, but not sense, transcripts in H2A.Z^WT^ mESCs, whereas depletion of H2A.Z alone had minimal effect on overall transcript levels (Figure 3B; Figure S5B). Notably, loss of H2A.Z suppressed the increase in antisense transcripts observed in the exosome mutants to wild-type levels. In contrast, Nanog and Tubb5, which lack significant promoter enrichment of H2A.Z (particularly at the −1 position), exhibited an increase in divergent ncRNA expression upon exosome depletion, but this expression was not suppressed by loss of H2A.Z (Figure 3B). Together, these data suggest that H2A.Z functions in concert with the nuclear exosome to regulate divergent ncRNA expression across eukaryotes.

### H2A.Z Cooperates with the Exosome to Repress a Subset of ncRNAs

**Figure 4:**
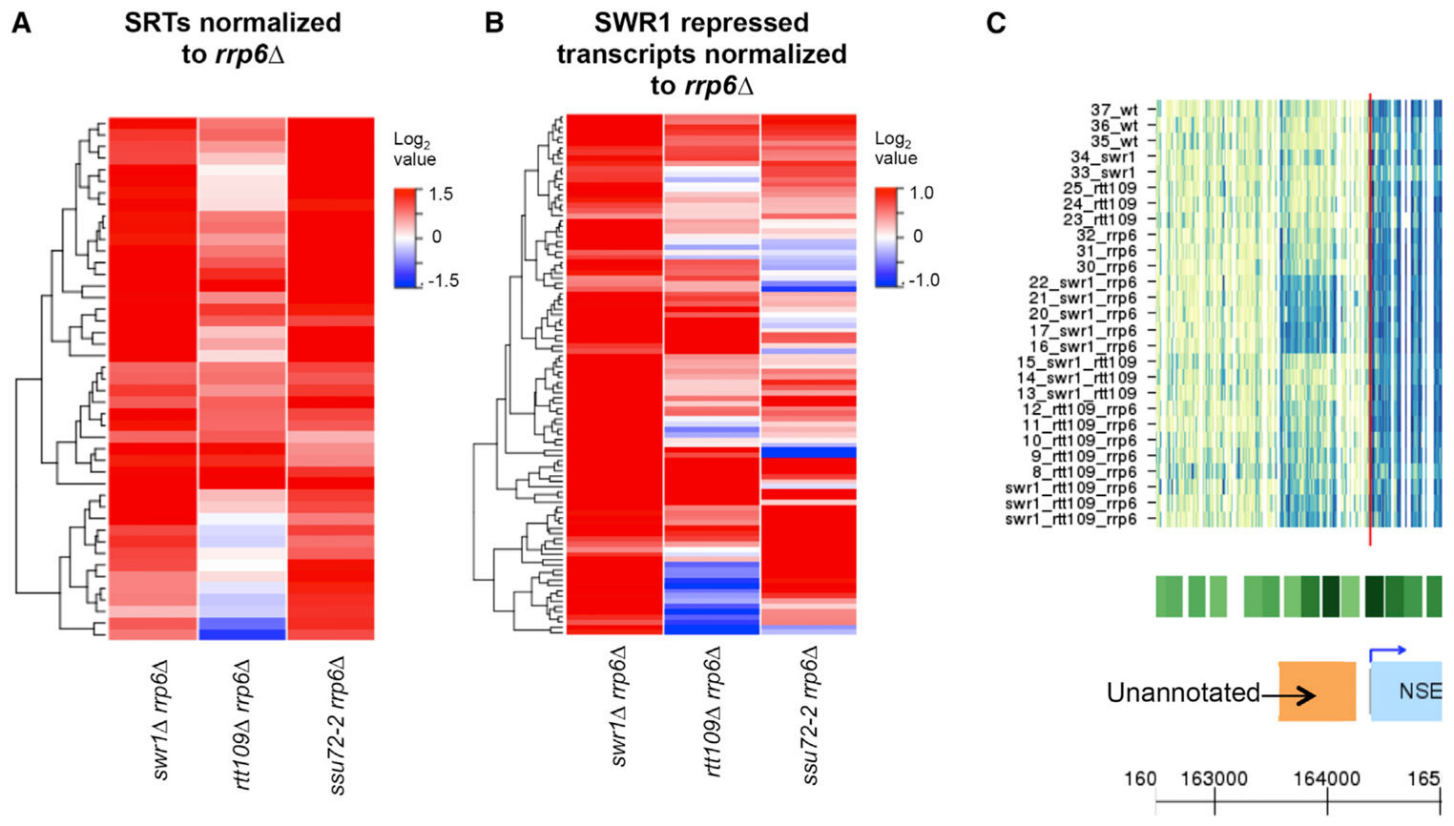
H2A.Z Inhibits Two Classes of Transcripts Associated with NFR Regions (A) Heatmap of normalized RNA abundance for SRTs in the *swr1Δ rrp6Δ*, *rtt109Δ rrp6Δ*, and *ssu72-2 rrp6Δ* strains compared to *rrp6Δ* and clustered as in Figure 2D. Only SRTs that significantly upregulated in *swr1Δ rrp6Δ* compared to *rrp6Δ* (n = 45) were used for the analysis. See also Table S4. (B) Heatmap of normalized RNA abundance levels for SWR1 repressed transcripts observed in this study for the *swr1Δ rrp6Δ, rtt109Δ rrp6Δ* and *ssu72-2 rrp6Δ* arrays compared to their respective *rrp6Δ* and clustered as in Figure 2D. Transcripts that significantly upregulated in *swr1Δ rrp6Δ* compared to *rrp6Δ* (n = 100) were used for the analysis. See also Tables S3 and S4. (C) Tiling array heatmap with array replicates as rows illustrate an example of genomic transcription of a previously unannotated transcript observed in *swr1Δ rrp6Δ* adjacent to a gene promoter. The green boxes shown above the gene browser view represent nucleosome positions, with dark green marking well-positioned nucleosomes. For the complete genome, see http://steinmetzlab.embl.de/cgi-bin/viewPeterssonLabArray.pl?showSamples=data&type=heatmap&gene=CUT505 (bottom). See also Data S1B.

Previous genome-wide studies uncovered strong genetic interactions among *SSU72*, *RTT109*, *HTZ1* (encoding H2A.Z), and genes encoding subunits of the SWR-C remodeling enzyme (Collins et al., 2007; Fiedler et al., 2009). Indeed, we found that the *swr1Δ ssu72-2^ts^* double mutant exhibited a synthetic slow-growth phenotype, consistent with H2A.Z deposition functioning in the same genetic pathway as *SSU72* (Figure S1C). Since Ssu72 represses a specific class of ncRNAs—the SRTs—we asked whether H2A.Z or H3-K56Ac might also repress some ncRNAs. Consistent with the genetic interactions, the *swr1Δ rrp6Δ* double mutant showed a significant upregulation of a subset of SRTs (n = 45) by 1.5-fold or more (FDR < 0.1), whereas the *rtt109Δ rrp6Δ* double mutant had less of an effect (Figure 4A; Figure S1B; Data S1). To further investigate potential repression of ncRNAs by H2A.Z, we performed automated segmentation analysis followed by manual curation (Tan-Wong et al., 2012) to identify transcripts that were repressed by H2A.Z and the exosome. This analysis identified 100 transcripts that were not expressed in the wild-type or *swr1Δ* strain, but were significantly increased by 1.5-fold or more in the *swr1Δ rrp6Δ* mutant compared to the *rrp6Δ* strain (FDR < 0.1) (Figures 4B and 4C). Notably, most of these transcripts were not de-repressed in the *rtt109Δ rrp6Δ* double mutant, although a subset was expressed at low levels in the *rrp6Δ* single mutant (Data S1). The majority of these ncRNAs (59) were located within intergenic regions, whereas the remaining 41 transcripts appear to be 5′ or 3′ extensions of existing transcripts (Figure 4B; Table S3) (Fox et al., 2015). A subset of these unannotated ncRNAs was also derepressed in the *ssu72-2 rrp6Δ* strain, suggesting that they may be related to SRTs (Figure 4B). Thus, H2A.Z deposition promotes the expression of many CUTs and also functions to repress a distinct group of ncRNAs, including a subset of SRTs.

### H2A.Z Facilitates Formation of Chromosome Interaction Domains

**Figure 5:**
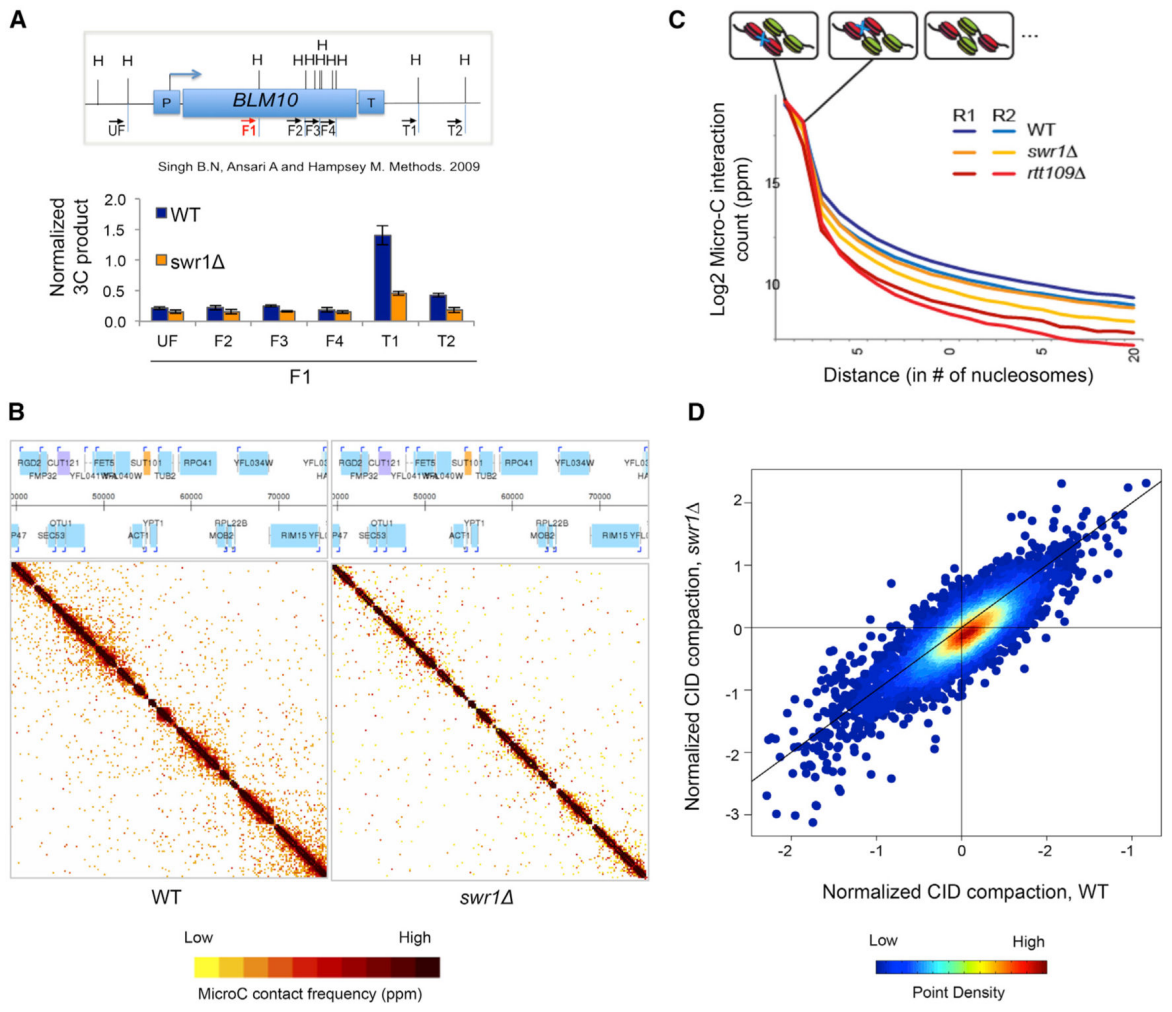
SWR-C Promotes Formation of Chromosome Interaction Domains (A) Chromosome conformation capture (3C) analysis of the *BLM10* locus (top: schematic) in wild-type (WT) and *swr1Δ* shows the frequency of interaction of each restriction fragment with the F1 fragment. Data are normalized to a control region on chromosome VI as the baseline contact probability. Error bars represent the mean of three biological replicates. See also Figure S6B. (B) Contact frequency matrix from Micro-C analyses for wild-type (left) and *swr1Δ* (right) for a region on chromosome VI with the gene annotations listed at the top. (C) Micro-C analyses show the log_2_ interaction count of one nucleosome with its successive neighboring nucleosomes in wild-type, *swr1Δ*, or *rtt109Δ* strains. (D) Density scatterplot for the compaction scores of chromosome interaction domains (CIDs) in the *swr1Δ* (y axis) compared to WT (x axis) (Kolmogorov-Smirnov test of the distributions yielded a p = 2.109e-15). The black line indicates x = y (no change).

Previous chromosome conformation capture (3C) studies suggested that Ssu72 functions as a “gene looping” factor and that this higher order chromosome structure may be key for repressing SRT transcription (Tan-Wong et al., 2012). Given the genetic and functional interactions between Ssu72 and H2A.Z, we tested whether H2A.Z might also regulate chromosome interactions that could underlie the repression of ncRNAs. First, we used 3C to monitor chromosome interaction frequencies at the *BLM10* locus, a known target of Ssu72-dependent gene compaction (Dekker et al., 2002; Singh et al., 2009). The 5′ and 3′ ends of *BLM10* exhibited far stronger interactions with one another than with intervening regions of this gene, consistent with localized gene compaction (Figure 5A). These enhanced interactions were lost in *swr1Δ*, indicating that compaction of this gene requires H2A.Z deposition (Figure 5A).

To ask whether H2A.Z affects genome organization at a global level, we used a modified Hi-C method, called Micro-C, to generate a high-resolution chromosome folding map for budding yeast. Micro-C has lead to the identification of abundant CIDs (Hsieh et al., 2015) which appear similar to mammalian topological-associated domains (TADs) (Dixon et al., 2012), although yeast CIDs are smaller (~5 kb) and contain an average of approximately one to five genes with strongly self-associating nucleosomes. Both transcriptionally active and repressed genes are found within CIDs, although highly transcribed genes are generally less compact than other genes in the genome. In our previous study, we reported that loss of H3-K56Ac results in diminished gene compaction (Hsieh et al., 2015). To test whether H2A.Z also contributes to this chromosome architecture, Micro-C analyses were performed on a *swr1Δ* strain. Interestingly, loss of H2A.Z deposition partially disrupted chromosome folding, consistent with a role for H2A.Z in CID formation (Figures 5B–5D). In particular, the loss of H2A.Z weakened the compaction of CIDs (Figures 5C and 5D), though the strength of boundary regions between CIDs remained largely intact (Figure 5B). Furthermore, loss of H2A.Z decreased compaction of the CID containing the *BLM10* gene, consistent with the 3C results, and even CIDs that lacked ncRNAs showed decreased compaction, consistent with a genome-wide defect in CID architecture that was independent of the transcriptional changes due to loss of H2A.Z (Figure S6D). Notably, the impact of H2A.Z on global gene compaction is less than either H3-K56Ac or Ssu72, consistent with the correspondingly weaker transcriptional defects due to loss of H2A.Z.

## DISCUSSION

H2A.Z and H3-K56Ac are hallmarks of dynamic nucleosomes positioned adjacent to promoters of protein-coding genes, but their impact on transcription has been enigmatic. Previous studies have shown that H2A.Z (Zhang et al., 2005) and H3-K56Ac (Williams et al., 2008; Xu et al., 2005) enhance the kinetics of transcriptional activation for highly inducible yeast genes, but they appear to play little role in the steady-state expression of most genes. Likewise, in mouse ESCs, H2A.Z is enriched at active and repressed gene promoters but depletion of this histone variant does not affect steady-state levels of active genes (Hu et al., 2013; Subramanian et al., 2013). Here, we identify functional interactions between these chromatin features and the RNA exosome, revealing a role for H2A.Z in the positive and negative regulation of ncRNAs and a general, activating role of H3-K56Ac on both ncRNA and mRNA transcription. Intriguingly, we find that H2A.Z along with H3-K56Ac and the CTD phosphatase, Ssu72, facilitates the formation of higher-order chromatin structures, called CIDs, suggesting that such structures may contribute to transcriptional control.

### Chromatin Dynamics Regulate ncRNAs

Many studies over the past few years have found that eukaryotic genomes are subject to pervasive transcription that produces an enormous number of ncRNA transcripts (van Dijk et al., 2011; Neil et al., 2009; Schulz et al., 2013; Tan-Wong et al., 2012; Xu et al., 2009). The steady-state level of many such ncRNAs are held in check by machineries that target these transcripts for their rapid degradation. For instance, divergent ncRNAs that occur at many bi-directional RNAPII promoters harbor binding sites for the Nrd1/Nab3 RNA binding complex that promotes both their termination and degradation by the RNA exosome (Schulz et al., 2013). Several recent reports indicate that chromatin structure can also repress ncRNA expression (Alcid and Tsukiyama, 2014; DeGennaro et al., 2013; Zofall et al., 2009). Buratowski and colleagues found that inactivation of the nucleosome assembly factor, CAF1, leads to increased expression of ncRNAs at many bidirectional yeast promoters (Marquardt et al., 2014). They suggested that assembly and/or stability of nucleosomes that occupy ncRNA promoters plays a key role in restricting their expression and reinforcing expression of the adjacent mRNA gene. Likewise, a recent study found that the esBAF chromatin remodeling enzyme represses expression of a large set of ncRNAs in mouse ESCs by positioning nucleosomes at ncRNA promoters (Hainer et al., 2015). Tsukiyama and colleagues have also reported that two yeast chromatin remodeling enzymes, RSC and INO80-C, inhibit expression of a large number of antisense ncRNAs in yeast (Alcid and Tsukiyama, 2014), and recently, we also found that INO80-C blocks ncRNA transcription within intragenic regions (Xue et al., 2015). How these enzymes prevent ncRNA expression is not yet clear, but a likely possibility is that they also enforce nucleosome positions that inhibit ncRNA promoter usage.

In contrast to mechanisms that inhibit ncRNA production, our results indicate that H3-K56Ac globally stimulates expression of divergent, promoter-associated CUTs in yeast. This stimulatory role for H3-K56Ac is consistent with a previous study indicating that nucleosome turnover can promote cryptic transcription within gene transcription units (Venkatesh et al., 2012). We also found that H2A.Z functions with H3-K56Ac to promote expression of a common set of CUTs in a non-redundant manner. Likewise, expression of divergent ncRNAs in mouse ESCs requires H2A.Z, and similar to the yeast CUTs, this correlates with H2A.Z levels at active divergent promoters. In general, these data suggest that H2A.Z and H3-K56Ac create a dynamic chromatin state that can facilitate expression of not only protein-coding genes, but also the adjacent ncRNA. Our study is consistent with a recent report that also identified a positive role for H2A.Z in CUT expression (Gu et al., 2015).

Genetic interactions between *SSU72* and H2A.Z led us to investigate roles for H2A.Z in repression of ncRNAs. Initially, we found that H2A.Z appears to function with the exosome and Ssu72 to repress expression of a subset of the SRT class of ncRNAs. In addition to the SRTs, we identified a group of 100 previously unannotated transcripts that were de-repressed in the *swr1Δrrp6Δ*strain. Interestingly, these transcripts are not detected in the *ssu72-2* single mutant, but a subset show increased expression in the *ssu72-2 rrp6Δ* strain compared to the *rrp6Δ* single mutant. As with SRTs, a subset (41) of these unannotated transcripts are 5′ or 3′ UTR extensions of existing ORFs. Furthermore, the aberrant 3′ extensions observed in the absence of *SWR1* occur primarily at convergent gene pairs, consistent with a previous report describing a role for H2A.Z in transcription termination in fission yeast (Zofall et al., 2009). Notably, the promoter regions that flank transcripts de-repressed in the *swr1Δ rrp6Δ* strain are depleted for H2A.Z compared to regions surrounding CUTs (Tan-Wong et al., 2012; Figure S4), suggesting that the repressive role for H2A.Z in this context may be indirect, or mediated through as yet unknown factors.

### Functional Interactions between Chromatin Dynamics and the RNA Exosome

**Figure 6:**
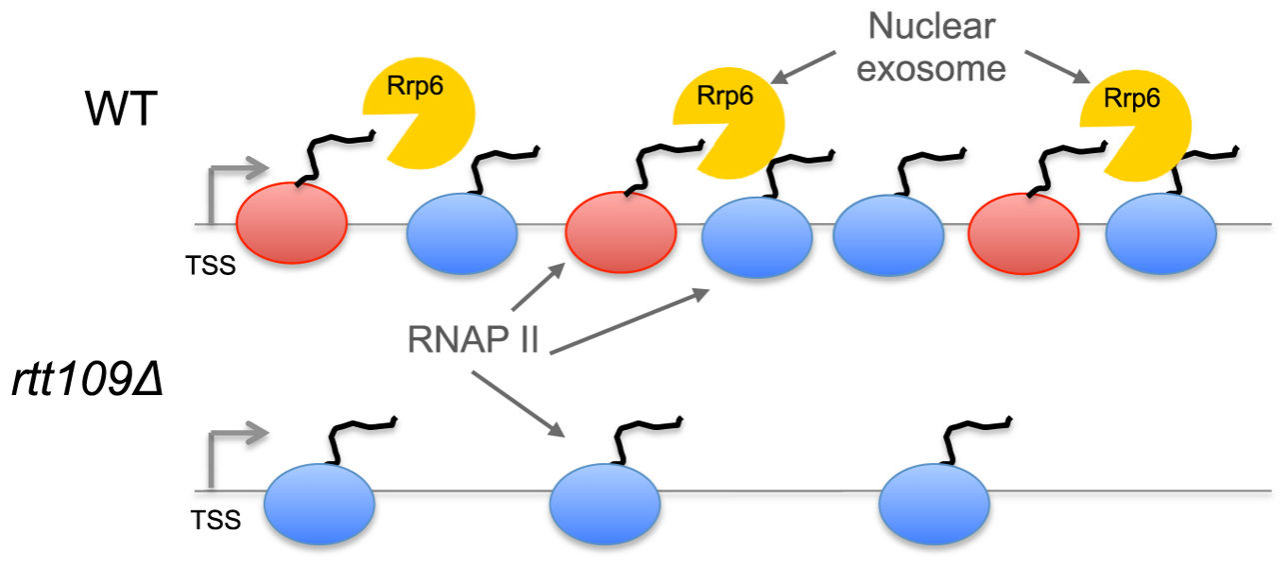
Model for How the RNA Exosome and Nucleosome Dynamics May Regulate Steady-State RNA Levels A model gene is shown in wild-type (WT) or *rtt109Δ* strains. In WT cells, a part of the population of elongating RNAPII molecules (red) are targeted by the RNA exosome (yellow) while the remainder RNAP II (blue) produce fully functional transcripts. In the absence of H3-K56Ac (*rtt109Δ*), RNAPII density is reduced, and the remaining RNAPII produces functional (blue) transcripts. Note that the RNA exosome may be present at both types of target genes, but its activity may only be apparent during cases of high RNAPII density.

Our RNA analyses identified 985 ORF transcripts that increased in abundance after inactivation of the nuclear exosome. This increase required H3-K56Ac, as these same transcripts were reduced in the *rtt109Δ rrp6Δ* double mutant. These data suggest that H3-K56Ac and the nuclear exosome act antagonistically at these ORFs to regulate their mRNA abundance. What is puzzling is that the steady-state levels of these ORF transcripts are not decreased in the *rtt109Δ* single mutant. Why does H3-K56Ac only seem to promote expression of these mRNAs in the absence of the exosome? One possibility is that each of these ORFs expresses two populations of transcripts: one type of transcript may be aberrant and be targeted for degradation by the exosome, and a second set may be functional (Figure 6). In this model, the decreased level of RNAPII, due to loss of H3-K56Ac, may favor production of functional transcripts and reduce formation of exosome-targeted transcripts (Figure 6, lower panel). For instance, fewer molecules of RNAPII may diminish the number of stalled, back-tracking RNA polymerases that are known to be targeted for exosome action (Lemay et al., 2014). Consistent with this view, ORFs whose transcripts increase in the absence of the exosome are enriched for both a high density of RNAPII and a high transcription rate (Figures S3C and S3D). This type of functional interdependency between RNAPII levels and exosome degradation may also underlie the regulation of divergent transcripts by H2A.Z and the exosome in mouse ESCs (Figure 3), as well as other cases where transcription and mRNA degradation appear to be linked (Haimovich et al., 2013; Sun et al., 2013).

### Chromosome Interaction Domains and ncRNA Transcription

Genome-wide, high-resolution analysis of yeast chromosome folding identified CIDs that encompass approximately one to five genes (Hsieh et al., 2015). The precise structure of these domains remains unknown, as 3C-based analyses find strong interactions between the 5′ and 3′ ends of genes (Figure 5A; Singh and Hampsey, 2007; Tan-Wong et al., 2012), whereas Micro-C instead recovers broader domains of interacting nucleosomes throughout gene bodies (Figure 5B). The technical reasons for this discrepancy remain unresolved—it seems likely that a pelleting step used in 3C may enrich for interactions between gene termini—but both CIDs and gene loops appear to unfold in *ssu72* mutants (Hsieh et al., 2015; Tan-Wong et al., 2012) and *swr1Δ* mutants (this study), suggesting that these assays provide distinct views of a common structure. Assembly of these compact domains requires subunits of the transcription Mediator complex (Med1), Rtt109 (H3-K56Ac), Ssu72, and H2A.Z. Of this group, only H2A.Z (and subunits of the SWR-C complex) shows negative genetic interactions with all three of the other regulators, *MED1*, *RTT109*, and *SSU72*, suggesting that it may be a key nexus for CID assembly or function (Collins et al., 2007; Fiedler et al., 2009).

A key question is whether CID architecture contributes directly to transcriptional regulation. The extent of gene compaction within CIDs anti-correlates with transcription, with highly active genes often localized either within or adjacent to strong boundary regions. In addition, strong boundaries are also enriched for CUTs, which are primarily divergent (Figure S6A). This suggests that boundaries between CIDs, which are generally associated with highly open and active promoters, may reflect chromatin domains that are generally permissive for transcription.

In contrast to boundary regions, highly compact genes within CIDs are transcriptionally derepressed in mutants that disrupt CID structure, suggesting that gene compaction within the CID architecture may help to promote or reinforce transcriptional repression. An inhibitory role for CIDs may be similar to the inhibitory “loop” mediated by H2A.Z between the promoter and the 3′ enhancer of the *CCND1* oncogene in mammalian cells (Dalvai et al., 2012, 2013). Likewise, the 3D organization of genes into CIDs may help to prevent expression of ncRNAs, such as SRTs and other ncRNAs that are repressed by H2A.Z. Consistent with thisview, we found thatSRTs are depletedfrom strong CID boundary regions (Figure S6A), and SRTs are derepressed when CIDs are disrupted in either the *ssu72-2* or *swr1Δ* strain. A role for CIDs in repression of SRTs provides an explanation for why a subset of SRTs is derepressed in the *swr1Δ* strain even though H2A.Z is not enriched at SRT promoters. Indeed, ncRNA transcripts that are repressed by H2A.Z are contained within CIDs that are more strongly de-condensed in the *swr1Δ* strain than CIDsharboringSRTsthatarenotrepressedbyH2A.Z(FigureS6C). An additional possibility that is consistent with the phenotype of *swr1Δ* and *ssu72-2* strains is that CID architecture may promote transcriptional fidelity by guiding correct sites of transcription initiation and termination, perhaps in part by localizing all of the machineries into a confined transcription domain. Thus, CIDs may generally reinforce normal transcription homeostasis, fine-tuning transcription of both coding and noncoding RNAs.

## EXPERIMENTAL PROCEDURES

### Yeast Manipulations and Standard Molecular Biology

All yeast deletion strains were made using standard procedures (Longtine et al., 1998) by tetrad dissection of heterozygous diploids (Amberg et al., 2005) in the W303 strain background (see Supplemental Experimental Procedures for a list of strains).

### Tiling Array and ChIP-Seq: Sample Preparation and Data Analyses

Yeast were grown in yeast extract peptone (YEP) media with 2% glucose at 30°C. Total RNA was prepared, labeled, and converted into cDNA by random primed retrotranscription of total RNAs as previously described (Castelnuovo et al., 2014) before being hybridized to Affymetrix tiling microarrays. At least three biological replicates for each genotype were analyzed from three independent array hybridizations. Each array was normalized using W303 genomic DNA as reference (Huber et al., 2006), and only transcripts scoring above a threshold background value were used for further processing, as previously published (David et al., 2006). Expression level for each transcript was estimated by the midpoint of the shorth (shortest interval that covers half the values) of the normalized probe intensities lying within the transcript as previously described (Xu et al., 2011), and differential gene expression analysis was performed using limma as detailed in Supplemental Experimental Procedures. Microarray data can be viewed on the Steinmetz lab browser (http://steinmetzlab.embl.de/peterssonLabArray/). qRT-PCR was used to validate the results of the tiling array (Data S2).

ChIP-seq samples were prepared (Watanabe et al., 2013) and analyzed either as in Teytelman et al. (2013) or by MACS2 (Zhang et al., 2008) as described in Supplemental Experimental Procedures. Two different biological samples were sequenced for each genotype. The 8WG18 antibody (Covance) was used for immunoprecipitations, as it is known to capture total RNA Pol II in genome-wide data (Bataille et al., 2012; Wong et al., 2014).

The complete annotation used in this publication is listed in Table S2. This study focuses on five major groups of significantly changed (p_adj_ = FDR < 0.1 and log_2_ fold change [LFC] > ± 0.59) transcripts defined below:

Group A ORFs are (1) significantly upregulated in *rrp6Δ* compared to WT and (2) reduced by > −0.59 LFC in *rtt109Δ rrp6Δ* compared to *rrp6Δ.* Refer to Figure 2D, Figure S4, and Tables S1 and S4.
Group B ORFs are (1) significantly downregulated in *rrp6Δ* compared to WT and (2) increased by > 0.59 LFC in *rtt109Δ rrp6Δ* compared to *rrp6Δ.* Refer to Figure 2D, Figure S4, and Tables S1 and S4. This group includes ORFs subject to transcriptional interference by adjacent CUTs.
Group C CUTs are (1) significantly upregulated in *rrp6Δ* compared to WT and (2) reduced by > −0.59 LFC in *rtt109Δ rrp6Δ* compared to *rrp6Δ.* Refer to Figure 2C, Figure S4, and Tables S1 and S4.
Group D CUTs are (1) significantly upregulated in *rrp6Δ* compared to WT and (2) reduced by > −0.59 LFC in *rtt109Δ rrp6Δ* as well as *swr1Δ rrp6Δ* compared to *rrp6Δ.* Refer to Figure 2C, Figure S4, and Tables S1 and S4. Up_ncRNAs are (1) significantly upregulated in *swr1Δ rrp6Δ* compared to *rrp6Δ* and include SRTs (n = 45), novel (n = 100), SUTs (n = 50), and CUTs (n = 29). Refer to Figure 4, Figure S4, and Tables S1 and S4. Unchanged_ncRNAs (n = 485) are SRTs that do not change significantly in *swr1Δ rrp6Δ* compared to *rrp6Δ.* Refer to Figure S4 and Table S1.

### Mouse Embryonic Stem Cell Culture and siRNA Treatment

H2A.Z^WT^ cells were generated as detailed previously (Subramanian et al., 2013), cultured in blasticidin (5 μg/ml) containing ESC media, and plated on blasticidin-resistant feeder cells (Iuchi et al., 2006). Depletion of exosome components Exosc5 and Exosc10 was performed by first plating H2A.Z^WT^ the absence of feeders on 10-cm plates the day before small interfering RNA (siRNA) treatment. DharmaFECT 1 reagent (Thermo Scientific) was used to transfect siRNAs against Exosc5 (Origene # SR406507) and Exosc10 (Origene # SR420984) in H2A.Z^WT^ ESCs (day 1) as per the manufacturer’s instructions. Day 2 post-transfection, doxycycline is removed from the cell media to generate siRNA-treated H2A.Z^KD^ ESCs. These cells are propagated in the absence of doxycycline and collected for RNA extraction on day 4 post-transfection. Control siRNA-treated H2A.Z^WT^ ESCs are propagated in the presence of doxycycline and collected for RNA extraction on day 4 post-transfection.

### RNA Extraction and RT-qPCR

RNA was extracted using Izol (5PRIME). Purified RNA was treated with DNase and purified using the RNA cleanup protocol in the QIAGEN RNeasy kit (QIAGEN). 5 μg DNase-treated RNA was reverse transcribed using SuperScript III (Invitrogen) and random hexamers according to manufacturer protocols. qPCR reactions were performed with SYBR Green Master Mix (LightCycler 480 SYBR Green I Master). Primer sequences are listed in Supplemental Experimental Procedures. Relative mRNA levels were quantified in triplicate for each transcript by the manufacturer’s software (Advanced Relative Quantification with Roche Lightcycler 480 Software Version 1.5) and using 28S rRNA levels for normalization.

### Micro-C and 3C Analyses

3C was done as in Singh et al. (2009). Micro-C was performed as in Hsieh et al. (2015) with three biological replicates each of the *swr1Δ* strain processed alongside three WT samples in order to minimize effects of batch variation.

## Supplementary Material

1

2

3

4

5

6

7

8

## Highlights

- H3-K56Ac and H2A.Z stimulate expression of promoter-associated, divergent ncRNAs
- Global transcriptional roles for H3-K56Ac are masked by the nuclear RNA exosome
- H2A.Z functions with the RNA exosome to repress expression of a subset of ncRNAs
- H3-K56ac and H2A.Z facilitate formation of chromosome interaction domains
