# Supplementary material for: Chromatin Dynamics and the RNA Exosome Function in Concert to Regulate Transcriptional Homeostasis
Source: Cell Rep. Author manuscript; Available in PMC 2015 Nov 30. (PMC4662874; doi:10.1016/j.celrep.2015.10.030)
Supplement: 1 [file NIHMS731410-supplement-1.pdf]

SUPPLEMENTAL INVENTORY:

Supplemental Figures, Data and Legends, S1-S6

Supplemental Data S1: Tiling array screenshots of different types of transcripts observed, related to Figures 2, 4

Supplemental Data S2: qRT-PCR confirmation of yeast tiling array data, related to Figure 2

Table S1: Summary of differential gene expression analysis, related to Figures 1, 2 and 4.

Table S2: Transcript annotation, related to Figures 2 and 4.

Table S3: SWR1 repressed transcript annotation and characteristics, related to Figure 4.

Table S4: Heatmap order and group definition, related to Figures 2 and 4.

Supplemental Information

Supplemental Experimental Procedures

Supplemental References
