# Supplementary material for: Chromatin Dynamics and the RNA Exosome Function in Concert to Regulate Transcriptional Homeostasis
Source: Cell Rep. Author manuscript; Available in PMC 2015 Nov 30. (PMC4662874; doi:10.1016/j.celrep.2015.10.030)
Supplement: 2 [file NIHMS731410-supplement-2.pdf]

Figure S1, Related to Figure 2 Rege et al.

A)

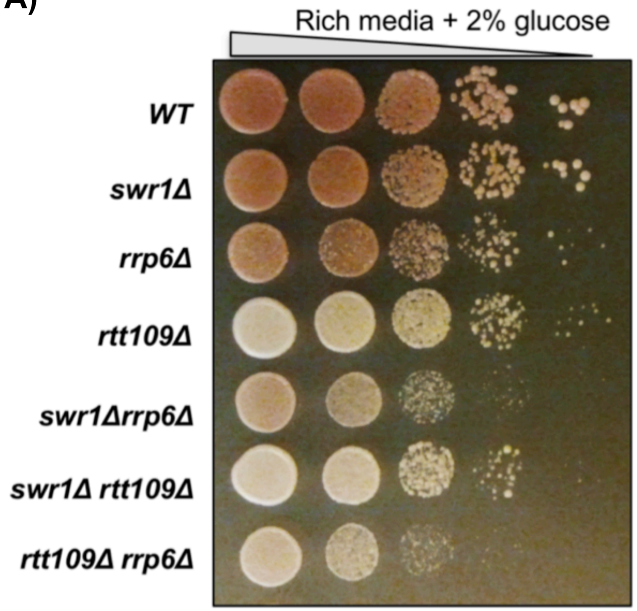

B)

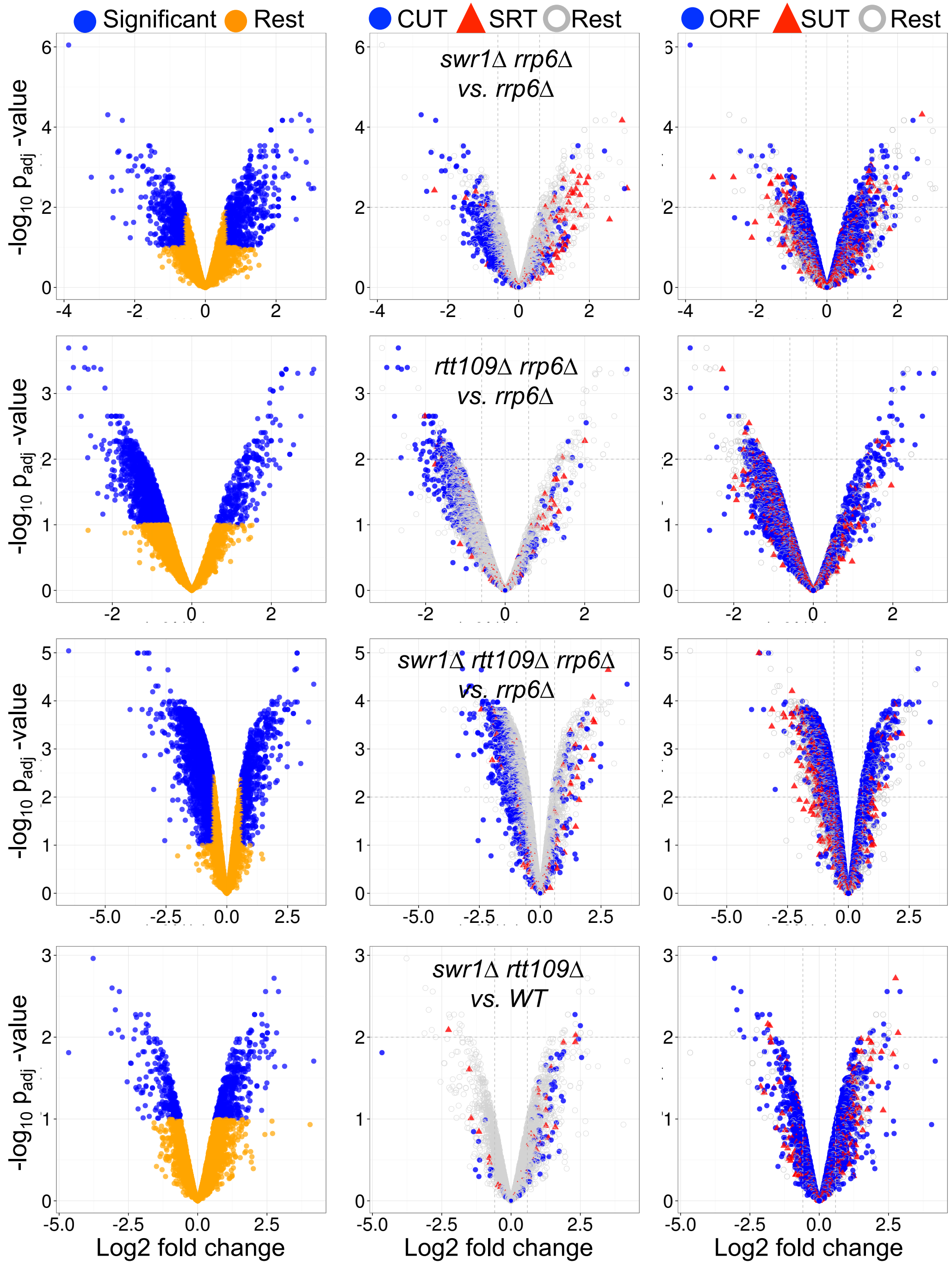

C)

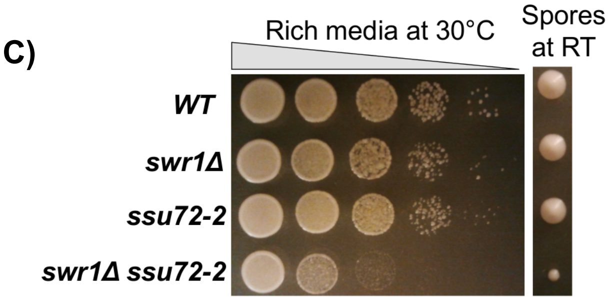

Figure S1: H2A.Z and H3-K56Ac affects various types of transcripts differently and H2A.Z interacts genetically with *SSU72.*

A) Synthetic growth defects of *swr1*Δ *rrp6*Δ and *rtt109*Δ *rrp6*Δ. Ten fold dilutions of each saturated overnight culture grown in YEPD media at 30°C was spotted onto plates of the same growth medium and incubated at 30°C for two days.

B) Volcano plots show the transcripts that change significantly highlighted in blue for each comparison (left most panels; p_{Adj} <0.1 and Log_2 Fold Change≥ ±0.59). Panels in the middle and on the right show volcano plots with two types of transcript colored differently in each panel for easy visualization.

C) Ten fold dilutions of each saturated overnight culture grown in YEPD media at 30°C was spotted onto plates of the same growth medium and incubated at 30°C. Note that the *ssu72-2* allele is a temperature- sensitive lethal, but grows at the semi-permissive temperature of 30°C.

Figure S2, Related to Figure 2 Rege et al.

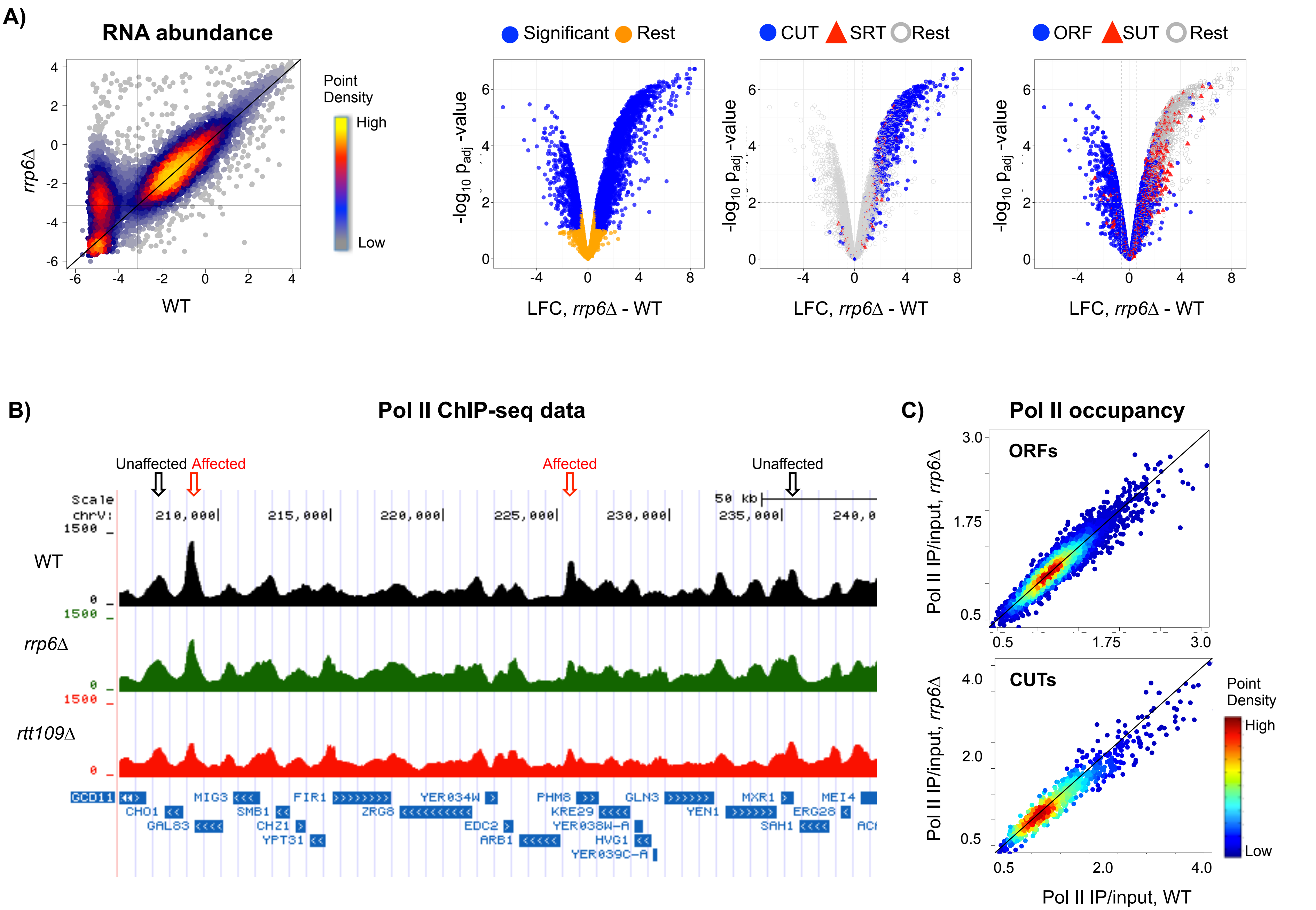

Figure S2: *rrp6Δ* does not affect Pol II occupancy but RNA abundance is increased.

A) RNA abundance measurements as in Figure 1A represented as density scatterplot (left panel). Volcano plots (right panel) show the transcripts that change significantly in the mutant compared to the wild type highlighted in blue (p_{Adj} <0.1 and Log_2 Fold Change≥ ±0.59).

B) Representative genome browser view of ChIP-seq analysis of Pol II for the wildtype (black), *rrp6Δ* (green) and *rtt109Δ* (red) normalized to the total library read count. The peaks marked as “Affected” and “Unchanged” were derived from analysis with MACS2.

C) Density scatterplots of Pol II IP/input values at coding regions of all ORFs (left) and CUTs (right) in the *rrp6Δ* compared to wild type. The black line indicates x=y (no change).

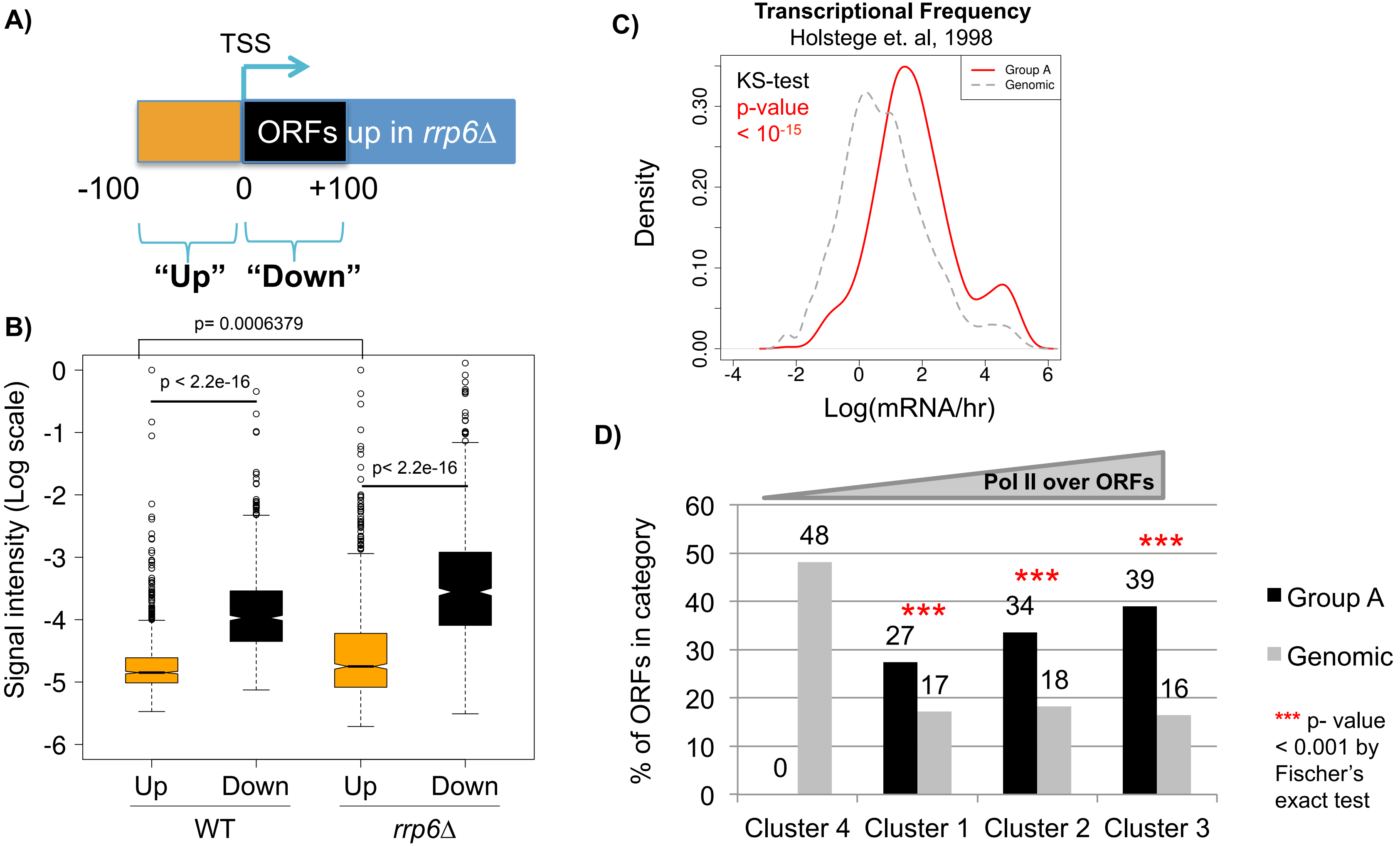

Figure S3: Defects in transcriptional termination do not account for Group A ORFs in the *rrp6Δ* and they are highly transcribed and have high Pol II density.

A) Schematic illustrating the upstream ("Up") and downstream ("Down") coordinates wrt the TSS of ORFs from which the signal intensity was extracted. The set of 985 ORFs upregulated in *rrp6Δ* was used for the analysis.

B) Boxplot for the two genotypes of the median signal over replicates is shown. Mann-Whitney-Wilcoxon Test was performed to compare the medians.

C) Density histogram distribution of transcriptional frequency data over the entire genome (Genomic, dashed) or over Group A ORFs (red).

D) Proportion of the entire genome (Genomic) or Group A ORFs that are members of one of four clusters defined by Venters and Pugh 2009. Cluster 4: no detectable Pol II; Cluster 1: Pol II primarily at promoter; Cluster 2: Pol II primarily at promoter and start of the ORF; Cluster 3: Pol II across the gene body, including 3' end.

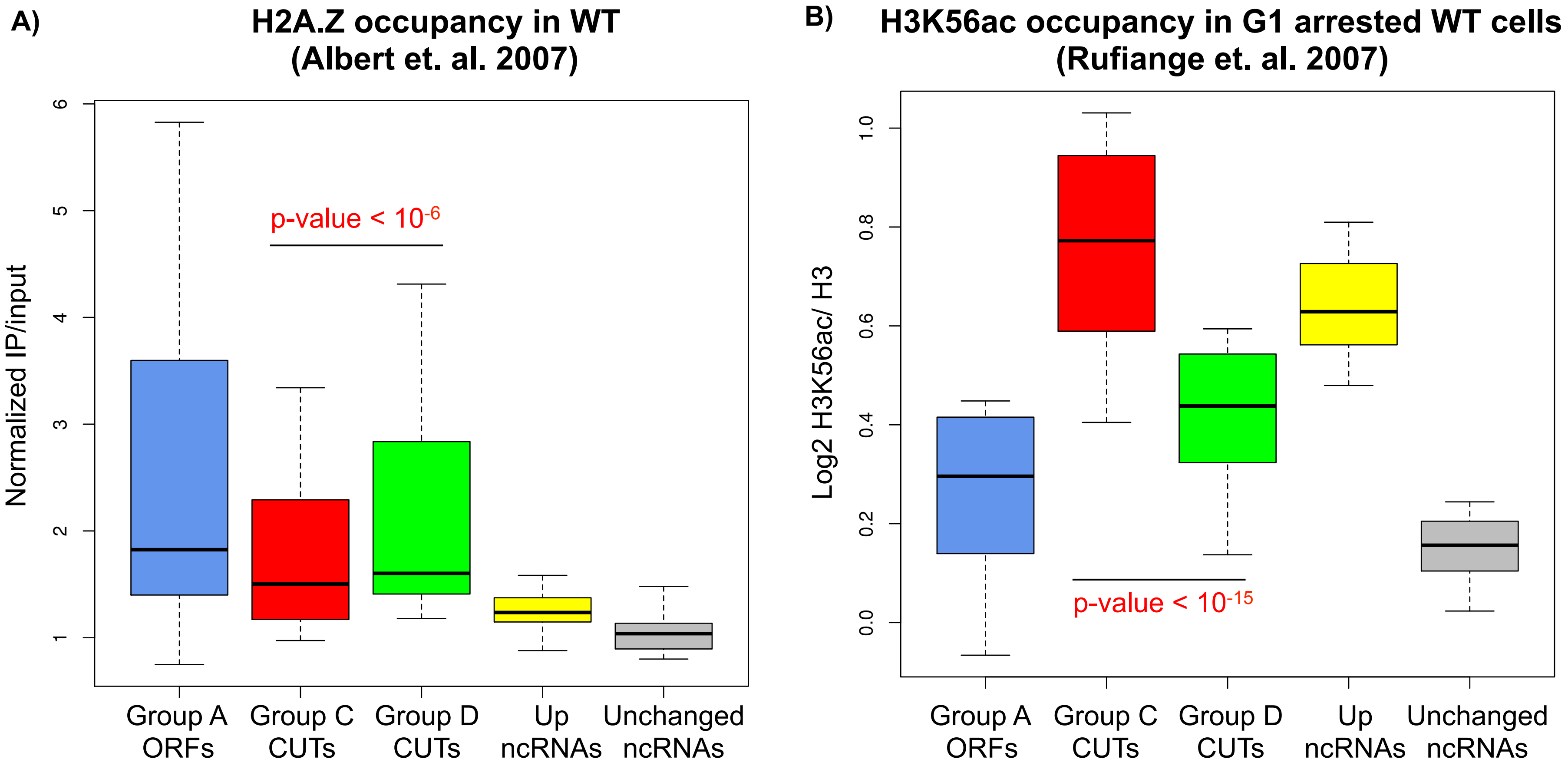

Figure S4: Distinct patterns of H3K56ac and H2A.Z are present at promoters of various transcripts

A) H2A.Z levels at Group A ORFs, Group C and D CUTs, Up_and unchanged ncRNAs. Two-sided KS test was used to compare medians of Group C and Group D CUTs. See Experimental procedures for definitions of each group.

B) H3K56ac levels at the same loci as in A. Two-sided KS test was used to compare Group C and Group D CUTs

Figure S5, Related to Figure 3

Rege et al.

A.

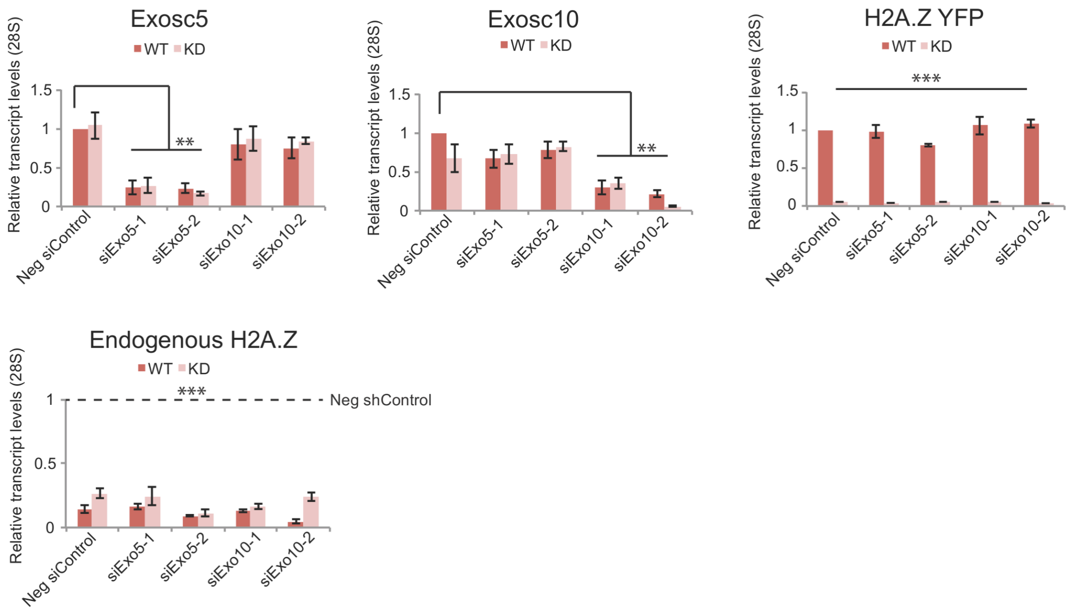

B.

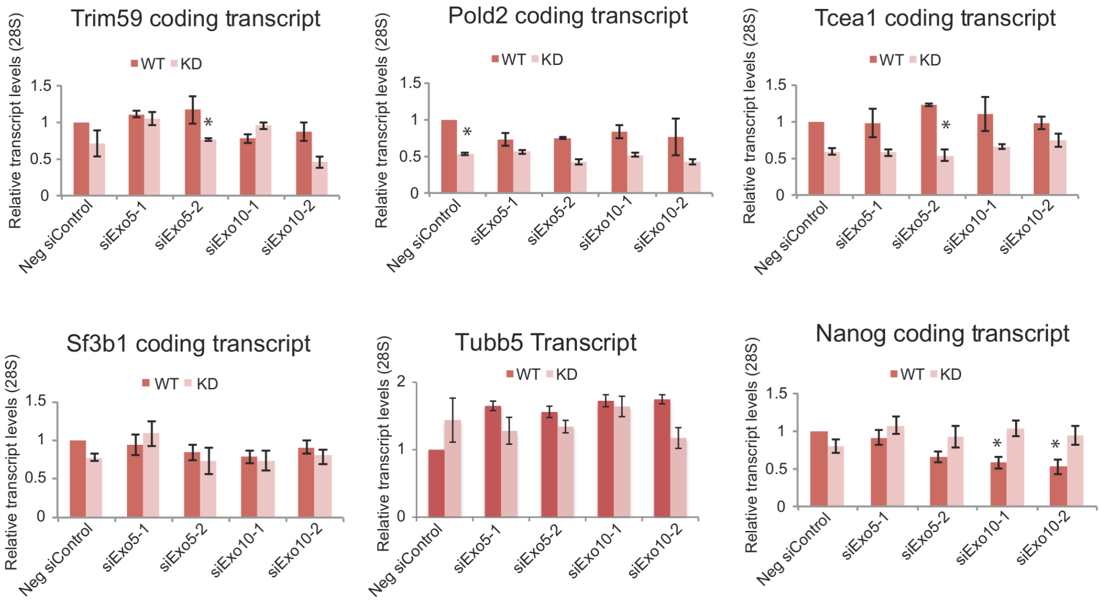

Figure S5: **siRNA-mediated depletion of Exosc5 and Exosc10 in H2A.Z^{WT} and H2A.Z^{KD} mouse ESCs does not affect sense coding transcripts**

A) qRT-PCR showing siRNA-mediated knockdown levels of Exosc5 and Exosc10 in H2A.Z^{WT} and H2A.Z^{KD} mESCs YFP- or H2A.Z 3'UTR-specific primers were used to measure the transgene and endogenous H2A.Z, respectively.

B) qRT-PCR showing the relative levels of sense full length transcripts in H2A.Z^{WT} (dark red) and H2A.Z^{KD} (pink) mESCs. Transcript levels were normalized to 28S rRNA levels and measured relative to transcripts in cells treated with non-specific siRNA (Neg siControl). Error bars represent standard deviations from a triplicate set of experiments.

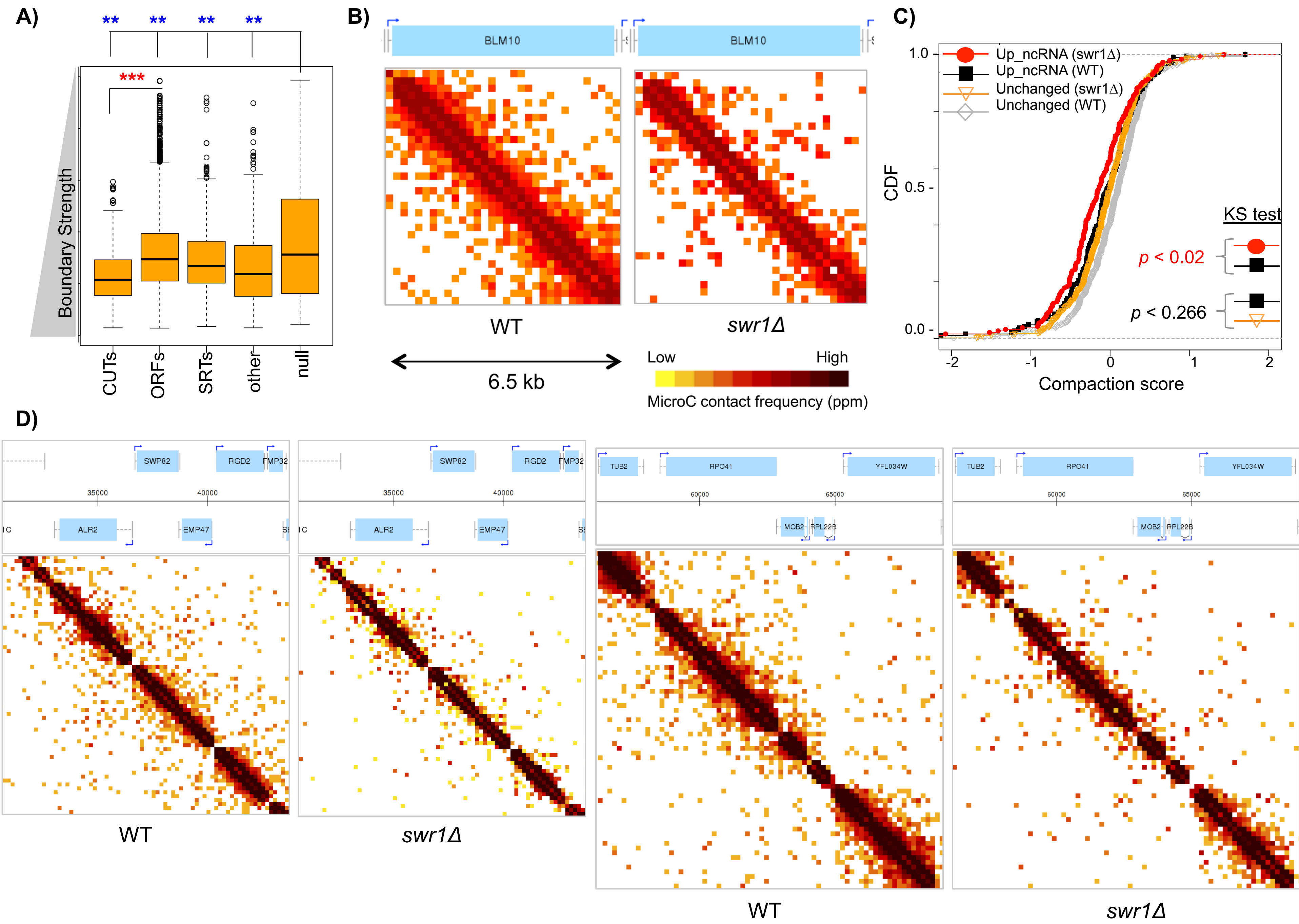
A)
**
***
Boundary Strength
CUTs
ORFs
SRTs
other
null
B)
BLM10
WT
swr1Δ
6.5 kb
Low
High
MicroC contact frequency (ppm)
C)
Up_ncRNA (swr1Δ)
Up_ncRNA (WT)
Unchanged (swr1Δ)
Unchanged (WT)
CDF
1.0
0.5
0.0
KS test
p < 0.02
p < 0.266
-2
-1
0
1
2
Compaction score
D)
SWP82
RGD2
FMP32
35000
40000
ALR2
EMP47
TUB2
RPO41
YFL034W
60000
65000
MOB2
RPL22B
WT
swr1Δ
WT
swr1Δ

Figure S6: Correlation of boundary strengths determined by Micro-C analysis with different genomic regions of interest
A) Box plot analysis of boundary strength associated with different loci. **(**)** Promoter proximal regions are associated with strong boundaries irrespective of transcript type. p-values were determined by Fischer's Exact Test. **(***)** CUT promoters are particularly enriched for strong boundaries, compared to other transcript promoters. Null are non-promoter NFRs. p-values determined by Fischer's Exact Test are all at least $p < 0.0001$.
B) MicroC data for the *BLM10* locus with the contact frequency of the individual nucleosomes in WT and *swr1*Δ.
C) Cumulative Distribution Function (CDF) of the compaction score for Up_ncRNA (n=269) and Unchanged_ncRNA (n=713) for *swr1*Δ and WT. Statistical significance of the two given distributions was tested using Kolmogorov–Smirnov test (KS test) using R.
D) MicroC data for select genomic loci devoid of ncRNA transcripts with the contact frequency of the individual nucleosomes in WT and *swr1*Δ.

Supplemental Data S1: Tiling array screenshots of different types of transcripts observed, related to Figures 2, 4

A) SRTs enriched in the *swr1Δ rrp6Δ;* B) SWR-C repressed transcripts found in this study and C) Transcriptional interference examples from Group B ORFs (see Figure 2C).

Supplemental Data S2: qRT-PCR confirmation of yeast tiling array data, related to Figure 2

Reverse transcriptase qPCR was performed on total RNA using SyBr Green. Melt curve was performed to ensure primer specificity and relative abundance is plotted for CUTs and ORFs. Error bars represent standard deviations from a triplicate set of experiments and differences tested using Student's T test.

Table S1: Summary of differential gene expression analysis, related to Figures 1, 2 and 4.

Each table contains the number of significantly expressed transcripts classified by transcript type for each limma comparison.

Table S2: Transcript annotation, related to Figures 2 and 4.

The complete list of transcript annotation. Column descriptions are below.

1) id
2) tx_ID (transcript ID)
3) seqName (chromosome)
4) strand
5) first (transcript start)
6) last (transcript end)
7) type (transcript category)
8) name
9) commonName
10) mappedType (confidently mapped transcript ends)
11) source (how the transcript ends were annotated)
12) isSRT (overlap with the SRT annotation in Tan-Wong et al., 2012)

Table S3: SWR1 repressed transcript annotation and characteristics, related to Figure 4.

The list of SWR1-repressed transcript annotation. Column descriptions are below.

1) seqName (chromosome)
5) first (transcript start)
6) last (transcript end)
4) strand
8) name
9) orientation (wrt neighboring ORF)
10) location_feature (5' or 3' extension of an ORF or a previously unannotated transcript)
11) limma_swr1rrp6.vs.rrp6 (classified as significantly different with an FDR < 0.1 and LFC > 0.59).

Table S4: Heatmap order and group definition, related to Figures 2 and 4.

The file contains four tabs describing the Groups described in Figure 2C, Figure 2D, Figure 4A, Figure 4B. Column descriptions as in Table S2.

Supplemental Information

Yeast strains

**Name**	**Genotype**
CY1089	HKY 579-10A Mata leu2-3, 112 his3-11, 15 ade2-1 ura3-1 trp1-1 can1-100 Rad5+ (W303)
CY1983	swr1Δ::HpH from CY1089
CY2071	rrp6Δ::NAT^R MATa, dissected from CY2052; spore 3B
CY2076	swr1Δ::$G418^R$ rrp6Δ::NAT^R, MATa, dissected from CY2052, spore 9C
CY2210	*rtt109Δ::HPH^R*, clone 23D segregant from CY2170
CY2211	swr1Δ::$G418^R$ rtt109Δ::HPH^R MATa clone 16D, segregant from CY2170
CY2212	rtt109::HPH^R rrp6Δ::NAT^R MATa clone 29c, segregant from CY2170
CY2213	swr1Δ::$G418^R$ rrp6Δ::NAT^R rtt109Δ::HPH^R MATa clone 29A, segregant from CY2170
CY2052	MAT a/α rrp6Δ::NAT in CY2031 clone 1
CY2031	MAT a/α CY1983 X CY927 swr1Δ/SWR1 in W303, Clone 1
CY927	W303-1B Mat α ade2-1 can1-100 his3-11,15 leu2-3,112 trp1-1 ura3-1

Oligonucleotides used in this study

**Name**	**Coding-Fwd**	**Coding-Rev**

Nanog	ATGCCTGCAGTTTTTCATCC	GAGCTTTTGTTTGGGACTGG
Trim59S	ATCCTGGACAAGAGGGTGTG	ATGCCCCGCTTCAAAGTTTT
Pold2	TCTTGAGGGTGCTTCGAACT	GCGGACCCTATGAATTTGCC
Tcea1	GATCGCAGGAGACTGGAAAG	GGGTTTCGATGGAACTCGTA
Sf3b1	TCTTGGCAAGCGAGGCACACTG	CAGAGCACTGATGGTCCGAACT
28S	GCGGAGAGCCGTTCGTCTTG	ACAAACCCTTGTGTCGAGGGC
CUT579	GCCGAATATTAGCTCCTTCG	TACATAATGCCAGCGACAGC
CUT848	AAACGGAGGTTTGTCACGTC	ACTTTTGCGGTTGCTCTCTC
CUT737	GCGCAAAAAGCTCAGTCTTG	ATCTGTCCCCGAATGGTATC
OLA1	AGAAGCCCGTGTTATTGTCC	GCATTACCCAAACCTTCACC
RPL36A	AGGGGTTTACCCCAAATACG	CTCTGGCTATTTCCATTGGTC
RRP45	GCAACACCAAAGTTCACTGC	CCTTCAAATGGCCTGTCTTC
CHRVIL	CATGACCAGTCCTCATTTCCATC	ACGTTTAGCTGAGTTTAACGGTG

**Name**	Antisense-Fwd	Antisense-Rev
Nanog	CCAATGTGAAGAGCAAGCAA	GTGGGGGATCTGAGTTTCCT
Trim59S	GTTTTCTGACCCAGCGTGTT	CGCAGGCTTCTTCCATTGTT
Pold2	GGATAGAGTGGGAGGGAAGC	CCCATCACTTAGCACTGGGT
Tcea1	CTATCCGGACTCGCGTTG	CTTTAAGCCCTCGGCAATG
Sf3b1	GCGGAAGAGGATGGCTACT	GTCTGTACAGCCCTGGCTTC

Pol II ChIP-seq Library sequencing depths

Strain	Total reads	Uniquely filtered mapped reads
Wildtype rep1	~7.8 Million	~7.5 Million
Wildtype rep2	~11 Million	~10.0 Million
rtt109Δ rep 1	~9.8 Million	~9.3 Million
rtt109Δ rep 2	~8.7 Million	~8.2 Million
rrp6Δ rep1	~11 Million	~11.0 Million
rrp6Δ rep2	~11 Million	~10.0 Million
Input	~29 Million	~28.0 Million

SUPPLEMENTAL EXPERIMENTAL PROCEDURES

Differential gene expression analysis for tiling array data and corresponding plots

Statistically significant transcripts between the mutants compared to either the *rrp6Δ* or WT were scored using the limma package (Smyth, 2004) in RStudio (R Core Team (2015). Appropriate model matrices were generated to apply the lmfit model to the data, following which eBayes statistics was implemented. Varying combinations of thresholds for p_{adj} (<0.1, <0.05) and Log_2 Fold Change (LFC) (> ±0.59, > ±1.0) were tested to confirm the results were not specific to a given condition. All detectable transcripts in the tiling array data that have a p_{adj} = FDR < 0.1 and Log_2 Fold Change (LFC) > ±0.59 were defined as statistically significant for a given comparison. The number of transcripts that change in each comparison is summarized in Table S1. Volcano plots show the $-\log_{10} p_{adj}$ value (Y-axis) obtained from limma analysis against the Log_2 Fold Change (X-axis) with transcripts with a p_{adj} =FDR < 0.1 and LFC > ±0.59 colored in blue and the rest in yellow and were made using ggplot.2 (Wickham, 2009). The scatterplots and heatmaps in Figure 2 were plotted as Log_2 ratios normalized either to wildtype (WT) or the *rrp6Δ* as indicated. heatmap.2 function from the gplots package was used for hierarchical clustering without any additional scaling of data (Warnes et al 2015). The details of the dissimilarity matrix calculation method (euclidean) and linkage agglomeration method (complete or median) are specified in the legends of each heatmap.

Transcript annotation and categorization

Transcript annotations originally defined for CUTs, SUTs, ORFs (ORF-Ts) and other

were obtained from (Xu et al., 2009) and combined with the SRT annotation from (Tan-Wong et al., 2012) to get a comprehensive set of known annotations. Additional segment features were further searched in the absence of *SWR1* and *RRP6* using the automatic segmentation algorithm with default parameter (Huber et al., 2006). Two criteria were used to focus on previously unannotated transcripts of interest to this study: 1) the transcript did not overlap with the known annotation set and 2) transcript abundance in the *swr1Δ rrp6Δ* was greater than that in the *rrp6Δ*. Although we cannot determine the precise origin of these transcripts from tiling arrays, we classified them as 5' or 3' UTR extensions of existing ORFs, rather than 'novel' transcripts, if the tiling array signal was obviously contiguous with and of the same intensity as the corresponding ORF.

The complete annotation used in this publication is listed in Table S2. The annotation of SWR1 repressed transcript and their characteristics are listed in Table S3. The corresponding signal intensity values are available as processed data in the GEO subseries GSE73110.

Pol II ChIP-seq

Yeast were grown in Yeast Extract Peptone (YEP) media (100ml per IP) with 2% glucose at 30°C and resuspended in breaking buffer (20% glycerol, 100mM Tris-Cl pH 7.5 and 1x PIC) with 600μl of silica/zirconia beads in a screw-cap tube. The cells were lysed with a bead beater (Biospec) for 6 cycles of 1 min each with one minute intervals on ice and cell breakage was confirmed microscopically. After a brief spin at maximum speed, NPS buffer (0.5mM spermidine, 1mM β-mercaptoethanol, 0.075% NP-40, 50mM NaCl, 10mM Tris-Cl pH 7.4, 5mM $MgCl_2$ and 1mM $CaCl_2$) was added to the chromatin prior to MNase (Worthington, 200 units) digestion at 37°C for 20 minutes and the reaction

stopped by addition of 24 mM EGTA while keeping the tubes on ice. The input fragment size distribution was confirmed to be in the range of 150-300bp by Bioanalyzer. The chromatin was immunoprecipitated with 6ul of 8WG16 Pol II antibody (Covance) or with no antibody (input control) and the DNA was purified by phenol-chloroform extractions and ethanol precipitation. IP samples were confirmed by RT-PCR and sent to BGI, China for library preparation and single-end Illumina sequencing (Hi-seq 2000). Library preparation was done using a standard BGI protocol as follows: i) quality control by Qubit and Agilent 2100 ii) Addition of A base to 3' end and adapter ligation iii) PCR amplification and size selection for 100-300 bp and, finally iv) Library QC by Agilent 2100 and qPCR. The number of reads obtained/ uniquely mapped from each library is listed in Supplemental Information.

ChIP-seq analysis pipeline

Fastq files were put through the FASTQC program before alignment to the sacCer3 genome using Bowtie2 to obtain SAM files (Andrews; Langmead et al., 2009; Li et al., 2009). Bowtie settings for mapping Pol II ChIP-seq reads (default preset in Galaxy) were -s 0 -u -1 -n 2 -e 70 -l 28 --nomaqround 10 -v -1 -k 1 -m -1 --maxbts 125 -o -1 --seed -1 SAMtools was used for SAM to BAM conversion with the FLAGs to discard PCR duplicates and multiple mapping reads. Each sample was normalized to total library read count (bamCoverage tool in the deepTools package) before displaying in the UCSC Genome browser. After determining strong correlation values, the replicates were summed for further analysis. The IP/input value for the corresponding transcript coordinates was calculated as described in Teytelman et al 2014 using BEDtools

(Quinlan and Hall, 2010). Similar results were obtained if TSS- proximal regions were included in the analyses (-200 and +200 bp relative to the Transcription Start Site (TSS) and Transcription Termination Site (TTS) respectively). Additionally, the data were also analyzed by MACS2 and the peaks determined as significantly different across WT and *rtt109Δ* (*bdgDiff* module) highlighted in the genome browser views (Zhang et al., 2008).

The IP/input values are available as processed data in the GEO subseries GSE72692.

Transcriptional Frequency of Group A ORFs

Data was downloaded from Holstege et. al, 1998 and used to plot the distribution of transcriptional frequency of Group A ORFs or the whole genome. Statistical significance of the two distributions was tested using Kolmogorov–Smirnov test (KS test) in R.

Pol II density/occupancy of Group A ORFs

Data was downloaded from Venters and Pugh, 2009 and used to find the proportional membership of Group A ORFs or the whole genome in the clusters defined in Figure 4 of Venters and Pugh, 2009. Statistical significance of the proportion was tested using a two-tailed Fischer's exact test. The characteristics of each cluster are summarized as follows:

Cluster 4: no detectable Pol II

Cluster 1: Pol II primarily at promoter

Cluster 2: Pol II primarily at promoter and start of the ORF

Cluster 3: Pol II across the gene body, including 3' end.

For more details see Figure 4A from Venters and Pugh, 2009.

Micro-C data was clustered using Java Treeview (Saldanha, 2004).

Supplemental References

Amberg, D.C., Burke, D.J., and Strathern, J.N. (2005). Methods in Yeast Genetics: A Cold Spring Harbor Laboratory Course Manual, 2005 Edition.

Andrews, S. FastQC A Quality Control tool for High Throughput Sequence Data. Http://www.bioinformatics.babraham.ac.uk/projects/fastqc/.

Eddelbuettel, Dirk (2013) Seamless R and C++ Integration with Rcpp. Springer, New York. ISBN 978-1-4614-6867-7.

Hadley Wickham (2011). The Split-Apply-Combine Strategy for Data Analysis. Journal of Statistical Software, 40(1), 1-29. URL http://www.jstatsoft.org/v40/i01/.

F C Holstege, E G Jennings, J J Wyrick, T I Lee, C J Hengartner et. al. (1998). Dissecting the regulatory circuitry of a eukaryotic genome. Cell Volume: 95, Issue: 5, Pages: 717-728.

Langmead, B., Trapnell, C., Pop, M., and Salzberg, S.L. (2009). Ultrafast and memory-efficient alignment of short DNA sequences to the human genome. Genome Biol. *10*, R25.

Lawrence M, Huber W, Pag\`es H, Aboyoun P, Carlson M, et al. (2013) Software for Computing and Annotating Genomic Ranges. PLoS Comput Biol 9(8): e1003118. doi:10.1371/journal.pcbi.1003118

Li, H., Handsaker, B., Wysoker, A., Fennell, T., Ruan, J., Homer, N., Marth, G., Abecasis, G., and Durbin, R. (2009). The Sequence Alignment/Map format and SAMtools. Bioinformatics *25*, 2078–2079.

Martin Morgan, Herv\'e Pag\`es, Valerie Obenchain and Nathaniel Hayden (). Rsamtools: Binary alignment (BAM), FASTA, variant call (BCF), and tabix file import. R package version 1.20.2. http://bioconductor.org/packages/release/bioc/html/Rsamtools.htm

Quinlan, A.R., and Hall, I.M. (2010). BEDTools: A flexible suite of utilities for comparing genomic features. Bioinformatics *26*, 841–842.

R Core Team (2015). R: A language and environment for statistical computing. R Foundation for Statistical Computing, Vienna, Austria. URL http://www.R-project.org/.

Alok J. Saldanha. Java Treeview—extensible visualization of microarray data. Bioinformatics (2004) 20 (17): 3246-3248 first published online June 4, 2004 doi:10.1093/bioinformatics/bth349

Smyth, G.K. (2004). Linear models and empirical bayes methods for assessing differential expression in microarray experiments. Stat. Appl. Genet. Mol. Biol. *3*, Article3.

Venters, B.J., and Pugh, B.F. (2009). A canonical promoter organization of the transcription machinery and its regulators in the Saccharomyces genome. 360–371.

Warnes G.R, Bolker B. and Lumley T. (2015). gtools: Various R Programming Tools. R package version 3.4.2. http://CRAN.R-project.org/package=gtools

H. Wickham. ggplot2: elegant graphics for data analysis. Springer New York, 2009

Zhang, Y., Liu, T., Meyer, C. a, Eeckhoute, J., Johnson, D.S., Bernstein, B.E., Nusbaum, C., Myers, R.M., Brown, M., Li, W., et al. (2008). Model-based analysis of ChIP-Seq (MACS). Genome Biol. *9*, R137.
