# Supplementary material for: Chromatin Dynamics and the RNA Exosome Function in Concert to Regulate Transcriptional Homeostasis
Source: Cell Rep. Author manuscript; Available in PMC 2015 Nov 30. (PMC4662874; doi:10.1016/j.celrep.2015.10.030)
Supplement: 3 [file NIHMS731410-supplement-3.pdf]

A)

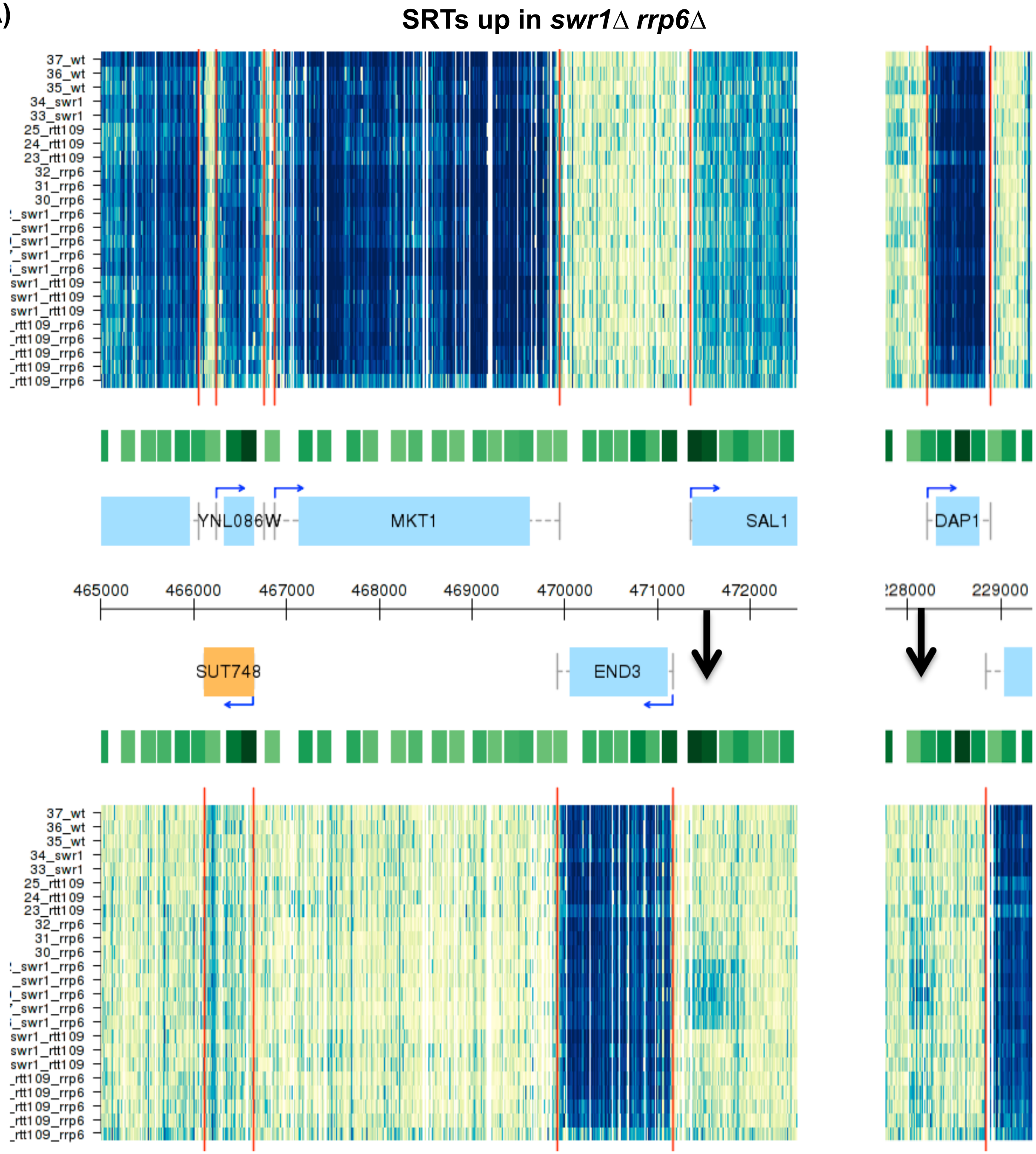
SRTs up in *swr1*Δ *rrp6*Δ
37_wt
36_wt
35_wt
34_swr1
33_swr1
25_rtt109
24_rtt109
23_rtt109
32_rrp6
31_rrp6
30_rrp6
YNL086W
MKT1
SAL1
DAP1
465000
466000
467000
468000
469000
470000
471000
472000
229000
SUT748
END3

B) **SWR-C repressed transcripts**

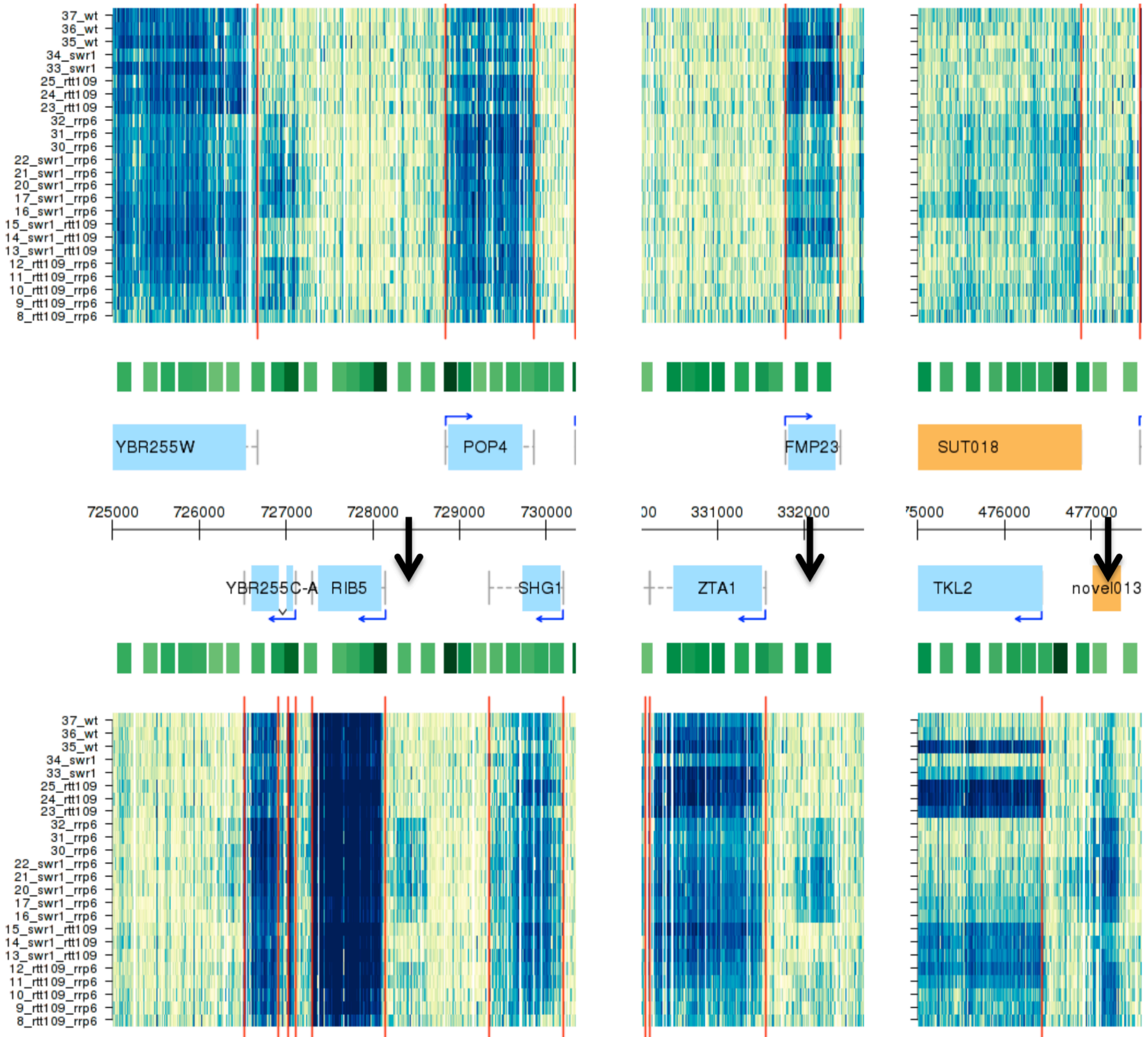

C) **Group B ORFs: Transcriptional interference examples in rrp6Δ**

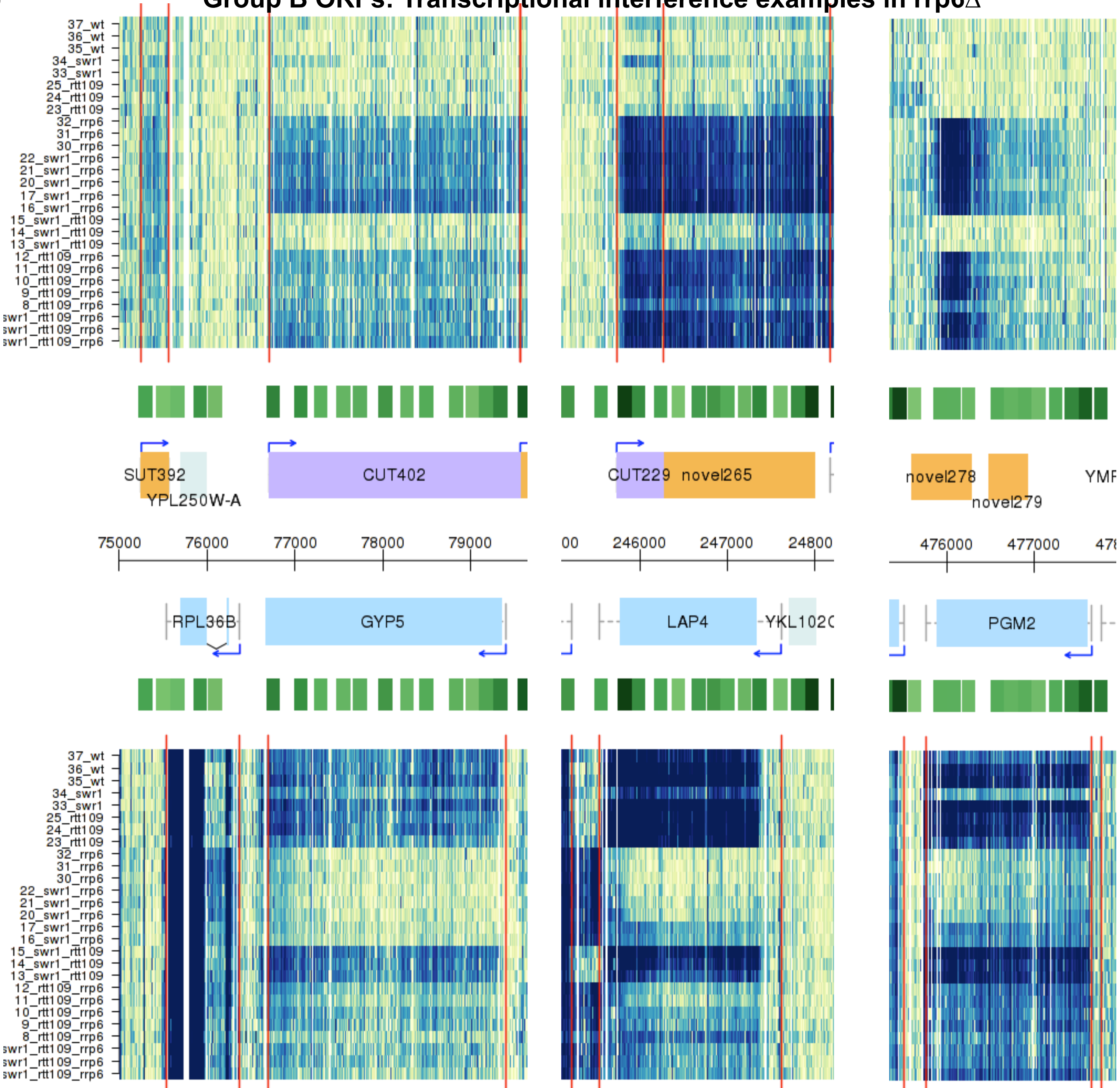

Supplemental Data S1: Tiling array screenshots of different types of transcripts observed. A) SRTs enriched in the *swr1Δ rrp6Δ;* B) SWR-C repressed transcripts found in this study (see Figure 4) and C) Transcriptional interference examples from Group B ORFs (see Figure 2C).
