# Supplementary material for: Chromatin Dynamics and the RNA Exosome Function in Concert to Regulate Transcriptional Homeostasis
Source: Cell Rep. Author manuscript; Available in PMC 2015 Nov 30. (PMC4662874; doi:10.1016/j.celrep.2015.10.030)
Supplement: 4 [file NIHMS731410-supplement-4.pdf]

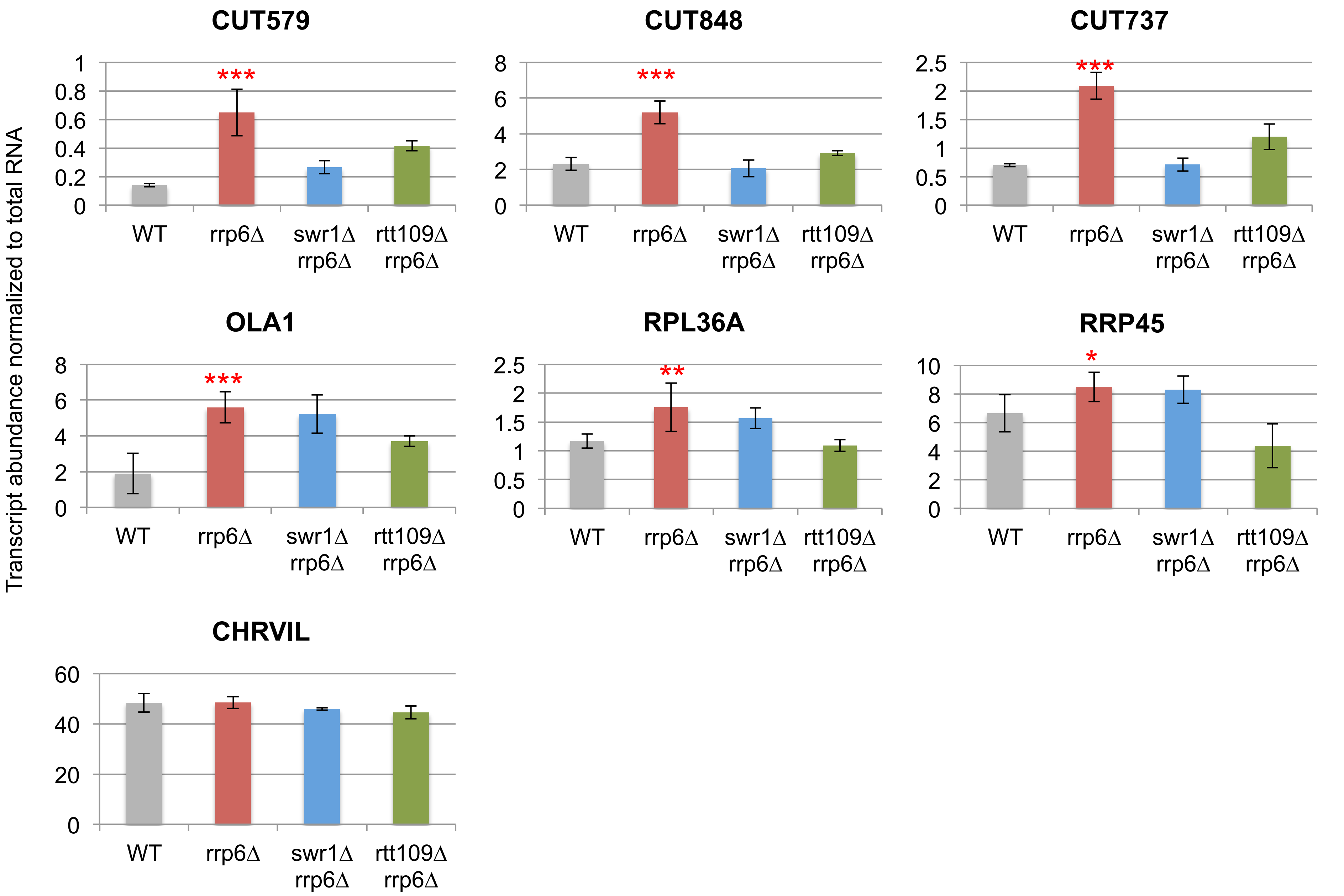
Transcript abundance normalized to total RNA
CUT579
1
0.8
0.6
0.4
0.2
0
***
CUT848
8
6
4
2
0
***
CUT737
2.5
2
1.5
1
0.5
0
***
OLA1
***
RPL36A
**
RRP45
10
*
CHRVIL
60
40
20
WT
rrp6Δ
swr1Δ rrp6Δ
rtt109Δ rrp6Δ

Supplemental Data S2: qRT-PCR confirmation of yeast tiling array data
Reverse transcriptase qPCR was performed on total RNA using SyBr Green. Melt curve was performed to ensure primer specificity and relative abundance is plotted for CUTs and ORFs. Error bars represent standard deviations from a triplicate set of experiments and differences tested using Student’s T test.
